# Subcortical modulation of the salience network during negative emotional processing in mood and anxiety disorders

**DOI:** 10.1038/s41380-025-03135-5

**Published:** 2025-08-28

**Authors:** Sevil Ince, Ben J. Harrison, Kim L. Felmingham, Alec J. Jamieson, Christopher G. Davey, James A. Agathos, Bradford A. Moffat, Rebecca K. Glarin, Trevor Steward

**Affiliations:** 1 Melbourne School of Psychological Sciences, The University of Melbourne, Parkville, VIC 3010 Australia; 2 Department of Psychiatry, The University of Melbourne, Parkville, VIC 3010 Australia; 3 The Melbourne Brain Centre Imaging Unit, Department of Radiology, The University of Melbourne, Parkville, VIC 3010 Australia

**Keywords:** Depression, Psychiatric disorders, Neuroscience, Psychology

## Abstract

Dysfunctional processing of negative emotional events is a key transdiagnostic feature of mood and anxiety disorders. This dysfunction is often associated with aberrant functioning of fronto-insular/cingulate regions involved in salience processing, including the anterior insula, dorsal anterior cingulate cortex, and ventrolateral prefrontal cortex (i.e., the salience network; SN). Coordination of SN responses to negative emotional events relies on bottom-up signals from subcortical regions commonly implicated in abnormal negative emotional processing, such as the amygdala and periaqueductal gray (PAG). Here, we used dynamic causal modelling (DCM) to investigate interactions between the amygdala, PAG and SN during negative emotional processing in mood and anxiety disorders. Thirty-seven participants with mood and anxiety disorders (29 Female) and 37 age and sex-matched healthy controls completed an emotional oddball paradigm during ultra-high field 7-Tesla functional magnetic resonance imaging scanning. DCM results revealed shared bi-directional interactions between the amygdala and PAG, and the SN during negative emotional processing. Specifically, while healthy control participants exhibited an inhibitory influence from the PAG to anterior insula, this effect was not detected in participants with mood and anxiety disorders (0.34 Hz, posterior probability = 1.00). Leave-one-out cross validation revealed this effect was large enough to predict diagnostic status, negative affect, depression, and stress levels. Additional group differences emerged in modulatory amygdala-to-PAG (−0.55 Hz, posterior probability = 1.00) and intrinsic PAG self-inhibitory (0.15 Hz, posterior probability = 1.00) connections. Our work indicates that differences in PAG-inhibition of the anterior insula likely contribute to maladaptive salience attribution and affective response in mood and anxiety disorders.

Biased processing of negative emotionally salient events is hypothesized to contribute to the aetiology, maintenance, and prognosis of mood and anxiety disorders [1, 2]. Cognitive theories have postulated that this bias may be underpinned by heightened attentional reorientation to negative emotional information and impairments in disengaging from negative emotional content [1, 3]. In support of this notion, previous neuroimaging investigations in mood and anxiety disorders have demonstrated altered engagement of fronto-insular and cingulate regions during the processing of salient negative emotional stimuli [4, 5]. These alterations trans-diagnostically converge on regions involved in detecting and evaluating salient stimuli (i.e., the salience network; SN), including the anterior insula (aINS), dorsal anterior cingulate cortex (dACC) and ventrolateral prefrontal cortex (vlPFC; [6, 7]. Coordinated SN activity enables adaptive adjustments in the attentional, autonomic-interoceptive and cognitive responses to negative emotional stimuli [8–11]. However, the efficient organization of these responses relies on dynamic interactions with subcortical brain regions, such as the amygdala and periaqueductal gray (PAG; [12–14]. Identifying precise circuit level interactions between these subcortical regions and the SN during negative emotion processing holds the potential to provide novel mechanistic insight into the pathophysiology of mood and anxiety disorders.

The amygdala and PAG are key subcortical structures involved in implementing rapid adaptive changes in attention, autonomic state and behaviour in response to negative emotional events [14–16]. In implementing these responses, the amygdala and PAG receive and integrate inputs from ascending sensory pathways [17–19] and descending cortical projections [20, 21]. In return, they relay this information to the subcortex, sensory, limbic and association cortices, including aINS, dACC and vlPFC [22–25]. Indeed, converging neuroimaging evidence has shown increased functional connectivity between the amygdala, PAG and SN regions during processing of negative emotional stimuli such as negative emotional images and threat cues [26–28]. Specifically, the amygdala has been implicated in amplifying the salience of negative emotional events to enable upstream access to attentional resources [29, 30] via its projections to SN regions [24, 31, 32]. Unlike the amygdala, the PAG is primarily involved in coordinating dynamic adjustments in autonomic-arousal and behaviour in response to negative emotionally salient events [15, 19, 27, 33]. Particularly, PAG-mediated parasympathetic arousal facilitates neural gain in attentional processing [14, 34], and may involve inhibition of insular and cingulate activity [31]. This role of the PAG can be conceptualized within the predictive processing (i.e., active inference) framework of mood and anxiety disorders. According to this account, psychopathology is associated with deficits in attenuation of sensory information in the interoceptive domain [35]. This impairment is posited computationally in terms of a failure to down weight precision-weighted interoceptive prediction errors [36, 37]. This precision weighting refers to the relative reliability of incoming interoceptive signals over the anticipated state, wherein signals with high precision induce stronger influence over perception and subsequently elicit greater adjustments to autonomic state giving rise these signals [35, 38]. Accordingly, the failure in down weighting interoceptive prediction errors results in elevated salience attribution and false inferences about interoceptive bodily states, and their emotional causes [35, 37]. Physiologically, this impairment corresponds to a failure of cortical gain control, particularly in regions mediating interoceptive inference including the aINS and dACC [39]. The disrupted parasympathetic-sympathetic autonomic state resulting from descending influence of these high-precision interoceptive signals further impair gain adjustment of ascending interoceptive signals [35, 40].

Previous animal work has shown that the amygdala and PAG are involved in mediating anxiety responses to aversive events and threat cues [23, 41]. In human neuroimaging investigations, converging evidence has demonstrated increased amygdala co-activity and altered functional connectivity with SN in response to negative emotional stimuli in anxiety [42–44] and depression [4, 45, 46]. Similarly, greater activity in the PAG and SN has been observed in people with anxiety disorders [47], and linked to anxiety levels in healthy populations during threat processing [48]. Although limited work has investigated disruptions in PAG activity in depression [49], recent animal studies have demonstrated that chronic stress leads to depressive behaviour and decreased excitatory activity within the PAG [50, 51]. Together these findings indicate anomalous functioning and connectivity of the amygdala and PAG with the SN during negative emotion processing in mood and anxiety disorders. However, given the dynamic nature of network interactions supporting negative emotion processing [14], the precise influence of this abnormal subcortical activity on the SN remains unknown. This shortcoming can be addressed by utilizing dynamic causal modelling (DCM), which allows for the testing of models to infer the directional influence of one region on another (i.e., effective connectivity) from functional neuroimaging data [52].

In the present study, we aimed to investigate differences in effective connectivity between the amygdala and PAG with the SN in individuals with mood and anxiety disorders compared to healthy controls. Following from our previous findings in healthy participants [31], we hypothesized that the PAG and amygdala would have inhibitory and excitatory influences on the aINS and dACC activity, respectively. We further hypothesized the amygdala would have an excitatory influence on the vlPFC to facilitate upstream access of negative emotional stimuli [32, 53] and for the vlPFC to have an inhibitory influence on the amygdala [54, 55]. Last, we hypothesized that group differences in effective connectivity would specifically emerge in bottom-up modulatory influences from the PAG and the amygdala. Additionally, we examined whether group differences in effective connectivity could predict diagnostic status and individual levels of psychopathology.

## Methods

### Participants

Forty-eight clinical participants with mood and anxiety disorders (i.e., clinical participants) were recruited to the study from the University of Melbourne Psychology Clinic and via the Research Experience Program at the Melbourne School of Psychological Sciences. All clinical participants underwent the Diagnostic Interview for Anxiety, Mood, and OCD and Related Neuropsychiatric Disorders (DIAMOND; [56] to confirm the presence of mood and anxiety disorders. Exclusion criteria for this group was the presence of psychosis, bipolar disorder, somatic symptom disorder, paraphilic disorder, obsessive-compulsive disorder, dissociative disorder, neurodevelopmental disorders, or substance use disorder. Exclusions due to low oddball count (*n* = 3; see *Experimental Paradigm* for details), excessive head movement during scanning (*n* = 3), and missing secondary (i.e., physiological) data (*n* = 5; see *Pre-processing* in Supplement for details), resulted in 37 clinical participants being included in the final sample (29 females, *M*_age_ = 22.27, *SD*_age_ = 5.51). Detailed clinical information on this sample is provided in Supplementary Table S1. The majority of the clinical participants (*n* = 22, 59.5%) met criteria for both an anxiety and depressive disorder.

Healthy control (HC) participants were recruited via online advertisements and consisted of 85 participants with no current diagnosis of a mental health disorder (screened using the Mini-International Neuropsychiatric Interview-7 [57]). Exclusions due to low oddball count (*n* = 1), excessive head movement (*n* = 2), and missing secondary data (*n* = 1), resulted in 81 participants (45 females) in this group. HC participants were one-to-one matched to the clinical participants according to sex and age, resulting in 37 HC participants (29 females, *M*_age_ = 22.51, *SD*_age_ = 4.67). It should be noted that none of the HC participants recruited for this study were included in our previous work [31].

Participants across both groups met the following eligibility criteria; (i) they were aged between 18–40 years, (ii) were fluent English speakers, (iii) had no MRI contradictions (e.g., metal implants, claustrophobia, pregnancy), (iv) had no major hearing impairments. All participants had normal or corrected-to-normal vision. All participants attended a single session at the Melbourne Brain Centre Imaging Unit (The University of Melbourne, Parkville). Furthermore, the 21-item Depression, Anxiety and Stress Scale (DASS; [58] was administered prior to the session to provide an overall measure of negative affect, as well as sub-scale scores of depression, anxiety, and stress. Sample characteristics are given in Table 1.

### Experimental paradigm

Participants completed the emotional oddball paradigm previously detailed in Ince and colleagues [31]. Participants were asked to identify a ‘target’ image in a stream comprising one frequently presented ‘standard’ image, and infrequent negative emotional and neutral distractor images. The standard image was of neutral valence and presented for 80% of all trials (240 presentations,) while the remaining trials consisted of infrequent oddball presentations (20 trials for each oddball category; target, negative emotional and neutral oddballs). Among these oddball images, the target was a single image of neutral valence. Remaining oddball trials consisted of trial-unique novel images with neutral valence (neutral oddballs) and negative emotional valence (negative emotional oddballs). All images were sourced from the Nencki Affective Picture System [59]; see Supplementary Table S2). A detailed description of the paradigm is presented in Supplementary Fig. S1 and Supplementary Methods. At the beginning of the task, participants were instructed to count the presentations of the target image throughout the task. To determine whether participants sufficiently paid attention during the task, at least 75% accuracy of the target oddball count (i.e., 15 out of 20) was used. Upon completion of the scanning session, participant ratings on arousal and valence of each image were collected on a 9-point Likert scale. To compare participants’ post-scan arousal and valence responses, independent samples t-tests and repeated measures ANOVA were conducted in IBM SPSS Statistics (version 29).

### Image acquisition, pre-processing, and general linear modelling (GLM)

Information regarding image acquisition and pre-processing, physiological noise correction and details of GLM analysis is provided in the Supplementary Methods. The primary contrast of interest was the direct comparison of negative emotional and neutral oddballs (NEG > NEU) to identify changes in brain activation associated with negative emotional salience response. Contrast images were estimated for each participant on the first level and entered to a second-level random-effects GLM using a one sample t-test, which was corrected for multiple comparisons using a whole-brain, voxel-wise false discovery rate (FDR) threshold (*p* < 0.05, K_E_ ≥ 10).

### Dynamic causal modelling (DCM)

#### Overview

DCM estimates directed neural population interactions (i.e., effective connectivity) which likely underpin observed neuroimaging data [52]. In estimating effective connectivity, DCM tests different combinations of a hypothesized functional network architecture in a Bayesian framework to find the model that best explains the observed neuroimaging data [52, 60]. It enables inferences to be made about intrinsic within-region and between-region connectivity (invariant to experimental changes) and modulation of these connections in response to experimental manipulations (i.e., during the presentation of salient images; [52, 61]. The estimated model parameters for between-region connectivity (measured in Hertz; Hz) represent the rate of change in activity in one region due to the influence of another region, where positive and negative connectivity parameters indicate the excitatory and inhibitory influence of one region over another, respectively [61]. The within-region (i.e., self-connection) estimates are unitless log-scaling parameters that multiply up or down the default value of self-inhibition (i.e., −0.5 Hz), and positive and negative values indicate increased and decreased within-region inhibition, respectively [61]. Self-inhibitory connections reflect excitatory-inhibitory balance in a region and determine the sensitivity of a region to the inputs from the rest of the network [62]. The estimated interactions within a DCM model can demonstrate both the influence of direct interactions within the modelled network or the combined influence of active neural populations between the nodes [63].

#### Model space and timeseries extraction

**Fig. 1:**
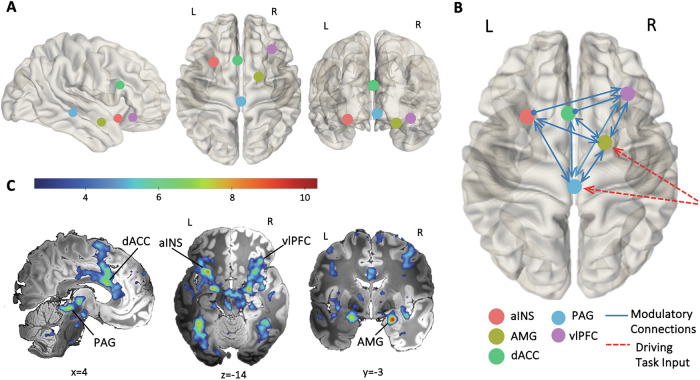
GLM results and DCM model space. **A** DCM model space node locations determined based on the peak values in the group-level NEG > NEU contrast GLM results for the left anterior insula (aINS; x = −29, y = 14, z = −14), right amygdala (AMG; x = 22, y = −3, z = −18), dorsal anterior cingulate cortex (dACC; x = −2, y = 16, z = 21), periaqueductal gray (PAG; x = 2, y = −32, z = −8), and right ventrolateral prefrontal cortex (vlPFC; x = 37, y = 29, z = −13). **B** Full DCM model depicting connections modulated by the negative emotional salience (blue lines) and driving task input (red dashed line). Render visualized using brainconn [105]. **C** Significant group-level GLM results for negative emotional salience (NEG > NEU). The group-level activation t-maps were overlaid on the ‘Synthesized_FLASH25’ (500 um, MNI space) ex-vivo template [106]. The colour bar indicates t-values. Comparisons were thresholded at voxel-wise *P*_FDR_ < 0.05, K_E_ ≥ 10. L, left; R, right.

The candidate model space consisted of five regions of interest (ROI); the PAG, amygdala, aINS, dACC and vlPFC (see Fig. 1A). ROIs were defined following the second-level GLM estimates for negative emotional salience (NEG > NEU) and based on our previous study using the same oddball paradigm [31]. Accordingly, the amygdala and vlPFC ROIs were on the right hemisphere, and the aINS ROI was on the left. Representative timeseries were extracted from each ROI at the subject level following published guidelines [61]. As such, the centre coordinate of the amygdala, aINS, dACC and vlPFC ROIs were identified by the subject-specific local maxima and constrained to be within the 8 mm of the group-level peak GLM result for NEG > NEU contrast (see Supplementary Fig. S2 for the distribution of subject-level peaks for each of these ROIs). Due to the anatomical size of the PAG and its proximity to adjacent midbrain regions and the aqueduct, the PAG timeseries were constrained to the active voxels within a PAG mask ([64]; see Supplementary Fig. S3 for a visualisation of this mask). One participant was excluded from further DCM analysis for not having their subject-specific local maxima in the aINS ROI within the 8 mm radius of the group-level peak GLM result for NEG > NEU contrast [61]. A detailed description for timeseries series extraction is provided in Supplementary Methods.

#### Model specification

Our candidate model space was specified using DCM 12.5. The task driving input was modelled into both the amygdala and PAG based on our previous findings [31]. The input matrix was mean-centred, therefore, the intrinsic connectivity represented the average connectivity across experimental conditions [61]. Intrinsic connections within the model were defined bidirectionally between all regions and included self-connections for each region. This connectivity architecture was informed by evidence from diffusion tractography and tract tracing studies in humans and non-human primates suggesting bidirectional structural connections between these regions [20–22, 25, 64, 65], as well as human neuroimaging studies showing both resting state and task-induced functional connectivity among these regions [26, 32, 66, 67]. The modulatory input by negative emotional salience was specified into feedforward and feedback connections between the PAG and amygdala, and aINS, dACC and vlPFC [68–70] and bidirectionally between the PAG and amygdala [19, 71]. The modulatory input was further specified into the connections from aINS and dACC to vlPFC [10]; see Fig. 1B).

#### Model estimation and inference

The full-model described above was fit to each participant’s time series (*spm_dcm_peb_fit*) to obtain posterior probability densities over the parameters of model connections (i.e., the probability of the model parameters given the observed timeseries [62]). Parametric Empirical Bayes (PEB) was used to estimate the group-level connectivity parameters. PEB is a hierarchical modelling approach, which consists of subject specific DCMs at the first level and a GLM of connectivity parameters at the second level [62]. The posterior probability densities of the model parameters in each participant’s DCM were re-estimated by *spm_dcm_peb_fit* function using the group average parameter estimates as their prior [62]. Re-estimated subject level parameter estimates were then taken to the second level and modelled, partitioning subject-level variance into shared group-level effects and additive random effects (*spm_dcm_peb*). Following PEB model estimation, Bayesian model reduction (BMR) was used to prune parameters that did not contribute to the model evidence (*spm_dcm_peb_bmc*). A Bayesian model average (BMA) was then calculated from these reduced models to obtain parameter estimates; while accommodating uncertainty over the model that best explains the data. A posterior probability (PP) > 0.99 was used to determine parameters with strong evidence (0.99 probability of parameter showing a non-zero effect, i.e., being present vs absent). In line with our aims, we investigated the effect of diagnostic status (clinical vs HC) while controlling for demographic variables (i.e., sex and age).

#### Leave-one out cross-validation (LOOCV)

We used leave-one out cross-validation (*spm_dcm_loo.m*) to explore whether the effect size of effective connectivity differences between clinical and HC participants were large enough to predict individual diagnostic status (clinical vs HC), as well as DASS scores. LOOCV iteratively fit a PEB model for all participants but one. The left-out participant’s group membership and DASS-scores were predicted from the effective connectivity parameters from the rest of the group. The significance of this prediction is indicated by a Pearson’s correlation between predicted and observed values.

## Results

### Demographic and behavioural results

No significant differences were observed between clinical and HC participants for age, sex, and educational level (Table 1). As summarised in Table 1, analysis comparing DASS scores between groups revealed that clinical participants had significantly higher scores than HC participants on the total DASS scale, as well as on depression, anxiety, and stress subscales. All DASS scores were significantly skewed (tested using Shapiro-Wilk, see Supplementary Table S3 for results) and were transformed using square root transformation to mitigate skewness (see Table 1). LOOCV with DASS scores on modulatory connections were ran using both transformed and non-transformed values for completeness.

Clinical and HC participants did not show significant differences in their target oddball count, post-scan arousal and valence ratings for negative emotional or neutral oddballs (see Supplementary Table S4). Overall, participants found the negative emotional oddballs more arousing, *F(1,72)* = 222.7, *p* < 0.001, and more aversive than neutral oddballs, *F(1,72)* = 917.06, *p* < 0.001. Diagnostic status did not have a significant main or interaction effect (results reported in Supplementary Table S4).

**Table 1:** Demographic and behavioural differences between healthy control and clinical participants.

	HC, *n* = 37	Clinical, *n* = 37	Group Comparison^a^		
			Statistic (χ^2^, *t* or *U*)	*p* Value	Effect size (*φ*, *d* or *r*^*2*^)
Sex, Female	29 (78.38%)	29 (78.38%)	χ^2^_1_ = 0.00	1.000	0.00
Age, Years	22.51 (4.67)	22.27 (5.51)	*t*_72_ = 0.21	0.838	0.05
Education, Years	16.31(2.29)	15.36 (2.43)	*t*_72_ = 1.72	0.089	0.40
Antidepressant Use	–	8 (21.60%)	–	–	–
DASS Total	7.92 (5.16)	29.68 (11.15)	*U* = 48.50	<0.001	0.64
DASS Depression	2.00 (1.78)	11.81 (5.43)	*U* = 36.00	<0.001	0.67
DASS Anxiety	1.84 (1.57)	7.27 (4.20)	*U* = 137.50	<0.001	0.48
DASS Stress	4.08 (3.02)	10.78 (4.24)	*U* = 136.50	<0.001	0.48
SQRT DASS Total	2.62 (1.03)	5.34 (1.07)	*t*_72_ = −11.10	<0.001	−2.58
SQRT DASS Depression	1.23 (0.72)	3.34 (0.82)	*t*_72_ = −11.79	<0.001	−2.74
SQRT DASS Anxiety	1.14 (0.75)	2.57 (0.82)	*t*_72_ = −7.88	<0.001	−1.81
SQRT DASS Stress	1.83 (0.88)	3.21 (0.68)	*t*_72_ = −7.62	<0.001	−1.77

Values are presented as *n* (%) or mean (*SD*).

*DASS* depression anxiety and stress scale [58], *HC* healthy control participants, *SQRT* square root transformed values.

^a^Categorical variables were assessed using χ^2^ with *φ* for the effect size. Continuous variables were analysed using independent samples *t*-tests with Cohen’s *d* for the effect size or using Mann-Whitney U tests with *r*^2^ for the effect size when the assumption of homogeneity of variance was violated.

### Group-level GLM results

Group-level GLM results showed that across both groups the processing of negative emotional salience (NEG > NEU) was associated with prominent activation in the SN, including our regions of interest, as well as mediodorsal, ventral anterior and ventrolateral thalamus, putative dorsal raphe nucleus, ventral tegmental area, substantia nigra and pons. Among our regions of interest, the aINS was bilaterally activated with its primary foci localized in the left ventral aINS along with a second separate peak in the dorsal aINS. The amygdala and vlPFC activations were also bilateral, with amygdala peak located in a region corresponding to the basolateral to centromedial nucleus in the right amygdala, while vlPFC peak was located rostrally in the right vlPFC (pars orbitalis). Additional significant activations were observed in the fronto-parietal, temporal and occipital regions (Fig. 1C; full list of significant activations in Supplementary Table S5). Peaks of activations in regions of interest were used to identify group-maxima for the ROIs in our model space (Fig. 1A; see Table S6 for peak t-values for regions of interest).

### DCM results

**Fig. 2:**
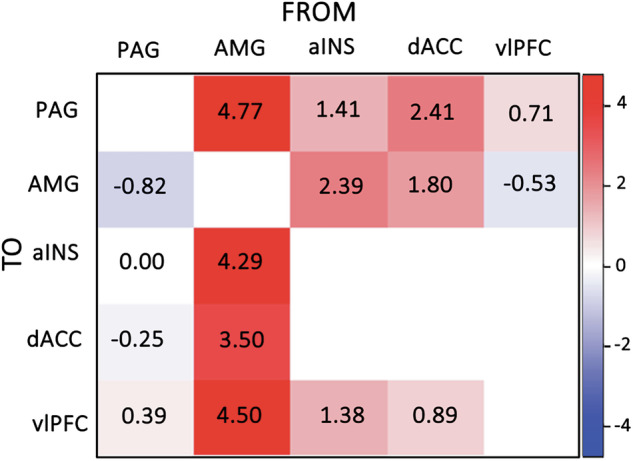
DCM modulation of effective connectivity by negative emotional salience. Heatmap depicts negative connectivity in blue and positive connectivity in red. The transparency of the heatmap indicates the strength of the modulation. aINS anterior insula, AMG amygdala, dACC dorsal anterior cingulate, PAG periaqueductal gray, vlPFC ventrolateral prefrontal cortex.

In the whole sample, bidirectional amygdala and PAG connections with the SN regions, along with connections from dACC and aINS to vlPFC showed evidence of modulation by negative emotional salience at a posterior probability (PP) > 0.99, apart from the PAG-aINS connectivity (Fig. 2; full results in Supplementary Table S7). During negative emotional salience processing, amygdala activity had a prominent bottom-up excitatory (positive modulatory) influence on the aINS, dACC and vlPFC and PAG activity. In return, it received relatively weaker top-down excitatory influence from aINS and dACC and a low inhibitory influence from the vlPFC. Likewise, the PAG received excitatory influence from the aINS, dACC and vlPFC. In contrast to the amygdala, the PAG exerted an inhibitory (negative modulatory) influence on the amygdala and dACC, along with an excitatory influence on the vlPFC. Lastly, aINS and dACC influence on the vlPFC was excitatory.

### Differences in effective connectivity between clinical and healthy control participants and leave-one out cross-validation

**Fig. 3:**
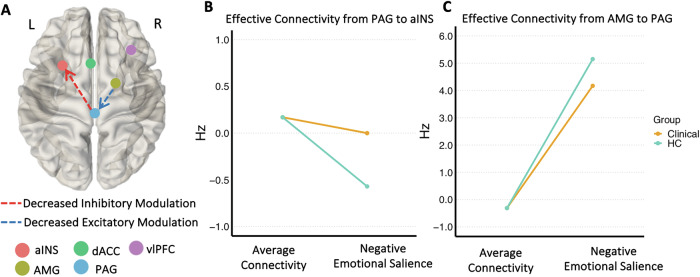
Differences between clinical and healthy control (HC) groups in directional interactions between amygdala, PAG and the salience network regions. **A** Differences in modulatory connectivity between clinical and HC that demonstrated strong evidence (posterior probability 0.99). Dashed red arrow indicates weaker inhibitory modulation, while blue dashed arrow indicates weaker excitatory modulation. **B** The change from average PAG-aINS connectivity (i.e., the context-independent coupling, left column) to modulation of PAG-aINS connectivity by negative emotional salience processing (right column), in clinical (orange) and HC (turquoise) groups. **C** The change from average AMG-PAG connectivity (left column) to modulation of AMG-PAG connectivity by negative emotional salience processing (right column), in clinical (orange) and HC (turquoise) groups. aINS anterior insula, AMG amygdala, dACC dorsal anterior cingulate, PAG periaqueductal gray, vlPFC ventrolateral prefrontal cortex. L, left; R, right.

**Fig. 4:**
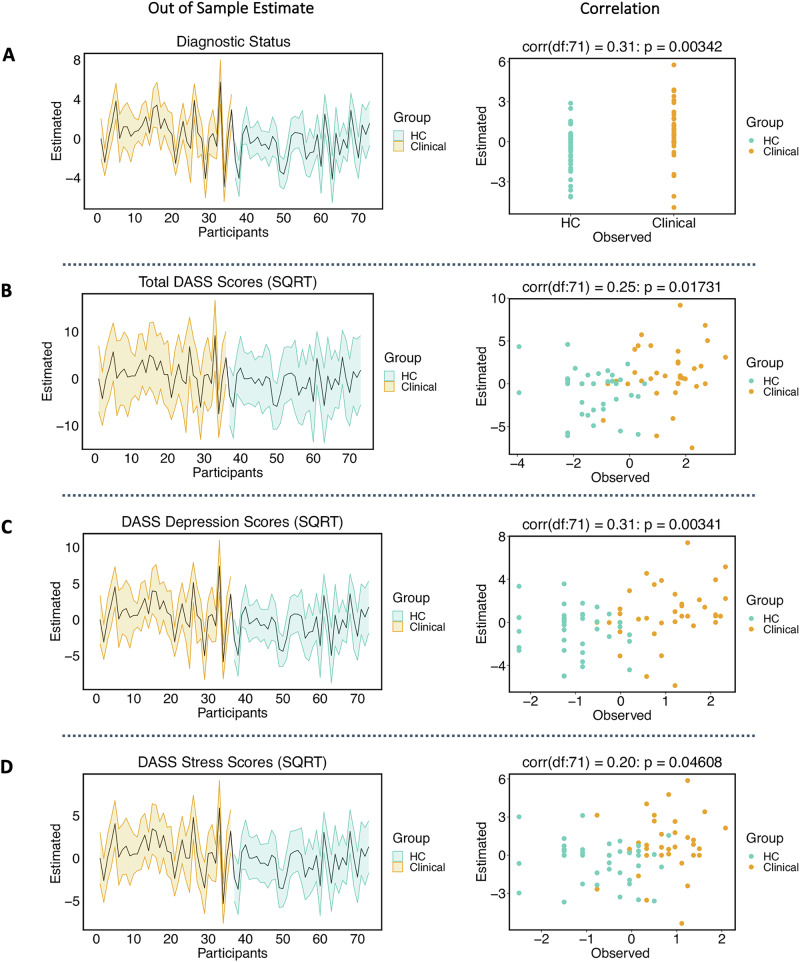
Leave-one-out cross validation of parametric empirical bayes (PEB) effects on PAG-to-aINS modulation during negative emotion processing. Orange depicts clinical and turquoise depicts healthy controls (HC) participants. Left: The out of sample estimates (black bold line) across participants with 90% credible interval (shaded area). Right: The correlation between observed and predicted values for **A** Diagnostic status (clinical vs HC), **B** mean-centred SQRT Total DASS scores, **C** DASS depression subscale scores, **D** DASS stress subscale scores. DASS Depression, Anxiety, Stress Scale. aINS anterior insula, PAG periaqueductal gray, SQRT square root transformed.

During negative emotional processing, clinical participants showed decreased inhibitory modulation from PAG to aINS (expected value = 0.34 Hz) and decreased excitatory influence from the amygdala to PAG (expected value = −0.55 Hz; Fig. 3; full details in Supplementary Table S8). LOOCV analysis was conducted to investigate whether the modulatory PAG-aINS and amygdala-PAG effective connectivity parameters could predict participants’ diagnostic status and DASS scores. For PAG to aINS modulatory connectivity, LOOCV results demonstrated a significant out-of-sample correlation between the predicted and observed diagnostic status (*r* = 0.31, *p* = 0.0034), total DASS scores (*r* = 0.25, *p* = 0.0173), DASS depression (*r* = 0.31, *p* = 0.0034), and DASS stress subscale scores (*r* = 0.20, *p* = 0.0461; Fig. 4). LOOCV results for this connection were not significant for DASS anxiety subscale scores (*r* = 0.09, *p* = 0.233, Supplementary Fig. S4). The size of the group-difference effect on amygdala to PAG modulation was not large enough to predict diagnostic status, or DASS scores (Supplementary Table S10 for all results, including results for non-SQRT transformed DASS scores).

**Fig. 5:**
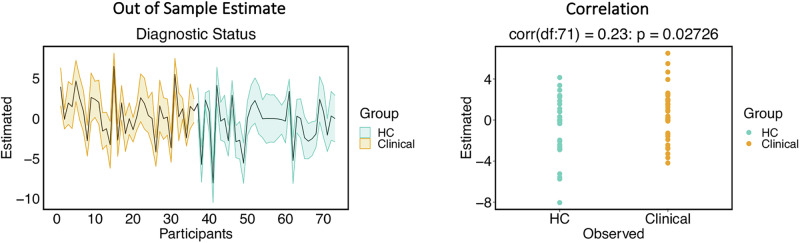
Leave-one-out cross validation of parametric empirical bayes (PEB) effects on intrinsic self-connectivity of the PAG. Orange depicts clinical and turquoise depicts healthy controls (HC) participants. Left: The out of sample estimates (black bold line) across participants with 90% credible interval (shaded area). Right: The correlation between observed and predicted values for diagnostic status.

Additional group differences between clinical and HC participants were observed in average effective connectivity (Supplementary Table S8 for full results). Among these, only self-inhibitory connectivity of the PAG (expected value = 0.15 Hz) resulted in a significant out-of-sample correlation between predicted and observed diagnostic status (*r* = 0.23, *p* = 0.02726; Fig. 5), but not with DASS scores. Full LOOCV analysis results for average connectivity are included in Supplementary Table S11. Age and sex were included across all analyses as covariates and effects for these are summarised in Supplementary Table S9.

## Discussion

Extending our previous work [31], the current study investigated dynamic causal interactions between the PAG and amygdala with the SN during negative emotion processing in mood and anxiety disorders. Our overall results confirmed significant bidirectional modulation between the PAG and amygdala, and the SN, which is consistent with previous human neuroimaging and animal work suggesting coordinated subcortical and cortical activity during processing of negative emotionally salient events [10, 69, 70, 72, 73]. We also identified novel group differences in bottom-up connectivity indicating that clinical participants do not exhibit PAG inhibition of the aINS as observed in HC participants during the processing of negative emotional stimuli. The size of this effect was large enough to predict diagnostic status, negative affect, depression, and stress levels. Clinical participants also showed increased baseline self-inhibitory connectivity within the PAG and decreased excitatory modulation of the amygdala to PAG connectivity. Of these, the former predicted diagnostic status. In contrast to our hypothesis, we did not observe any group differences in bottom-up amygdala connectivity.

### Bi-directional modulatory interactions between the amygdala and PAG, and the SN during negative emotional salience processing

We observed a significant excitatory influence from the amygdala to SN. In turn, the amygdala received excitatory influences from the dACC and aINS and an inhibitory influence from the vlPFC. Here, we modelled amygdala connectivity in the right hemisphere based on the greater right lateralized amygdala activation observed during processing of negative emotional salience. This is in line with right amygdala involvement in the rapid temporal processing of emotional stimuli, particularly in event-related designs, such as our paradigm [74]. The amygdala, specifically its basolateral aspect, receives rapid sensory inputs via subcortical routes [18, 75] and sends ascending projections to cortical sites including the dACC, aINS and vlPFC [24, 32]. Therefore, consistent with previous lesion [29, 30] and neuroimaging studies [32], this excitatory amygdala effect lends additional support to its role in upregulating negative emotional information to the SN to facilitate the detection and processing of negative emotional events [31]. The involvement of dACC, aINS and vlPFC have been commonly observed in response to negative emotional events [73, 76] and implicated in complementary but distinct roles during negative emotional salience processing [76, 77]. For instance, the dACC has been predominantly implicated in encoding contextual changes, and subsequently adjusting autonomic and motor responses [78, 79]. Supporting this, recent animal work has identified excitatory projections to the basolateral amygdala from the dACC which modulate motor responses to aversive events and have anxiolytic effects [69]. In contrast, the aINS incorporates sensory inputs with homeostatic/visceral information [80] and is involved in generating affective responses [81]. Similar to the dACC, the aINS sends excitatory projections to the amygdala that encode negative valence and modulate anxiety levels [70]. As hypothesized, we also observed an inhibitory influence from the vlPFC to the amygdala. This inhibitory vlPFC influence and right lateralization of group peak vlPFC response during negative emotional salience processing coincide with previous research demonstrating vlPFC involvement in inhibiting negative emotional distraction during cognitive tasks [54, 82] as compared to greater left vlPFC engagement and downregulation of amygdala activity highlighted in reappraisal-based emotion regulation studies [83]. In doing so, the right vlPFC integrates and compares contextual information provided by fronto-parietal regions with sensory inputs from subcortical regions, such as the amygdala [32] and salience information from the dACC and aINS [10].

In line with our hypothesis and previous findings [31], the PAG showed an inhibitory effect on the amygdala and dACC, and further exerted an excitatory influence on the vlPFC. The inhibitory influence of the PAG on the amygdala and dACC likely inhibits sympathetic activity and motor responses coordinated by these regions [9, 84, 85].This interpretation is consistent with animal work that has shown upon threat encounter PAG activity leads to cessation of ongoing behaviour and attentional reorientation for threat assessment with a concomitant increase in parasympathetic arousal [19, 33, 86]. Supporting human neuroimaging evidence has also demonstrated PAG involvement in increased parasympathetic autonomic arousal (indexed by reduced heart rate) and freezing responses to negative emotionally arousing images [27] and threat cues [15]. These PAG-mediated responses have been shown to enhance perceptual evaluation of threat and prepare organisms for further action [15, 34]. Furthermore, the PAG inhibition may have a role in the sensory attenuation of interoceptive signals and cortical gain control posited within the active inference account of interoception [39, 40]. This inhibitory PAG influence can be mediated via direct projections from the PAG to the (central) amygdala [71], and indirect pathways to the dACC, including thalamic nuclei [23, 87, 88].

Additionally, the PAG received an excitatory influence from the amygdala, dACC, aINS and vlPFC. The amygdala is one of the primary input regions to the PAG and integral in driving PAG-mediated responses to aversive/threatening events [19, 89]. The PAG also receives descending projections from aINS, dACC, and vlPFC [20], which exhibits increased connectivity with the PAG during threat processing [26]. Similar to the amygdala, these descending projections to the PAG signal changing environmental contingencies for flexible shifts in PAG mediated sensorimotor and autonomic responses. For instance, animal evidence has demonstrated that activation of dACC increases PAG activity and modulates approach and avoidance responses to pain and innate threat [68]. Overall, our findings provide evidence for a dynamic interplay of interactions between the amygdala, PAG and SN that can mediate attentional, autonomic and behavioural responses during negative emotional salience processing.

### Clinical participants show differences in PAG effective connectivity compared to healthy controls

Our findings revealed an inhibitory influence from the PAG on the aINS activity in HC participants. However, clinical participants did not demonstrate this inhibition, which is a novel observation in human neuroimaging studies. Furthermore, this effect was large enough to predict diagnostic status, as well as negative affect, and depression and stress symptoms. Afferent autonomic (baroreceptor) signals in the aINS are a key source of information for the cortical representation of physiological arousal and crucial for shaping affective responses to external stimuli [80, 90]. However, these interoceptive signals impair the perception of external stimuli [80], which is abolished with aINS lesions [91], indicating the aINS integrates visceral interoceptive signals with salient external information. An opposite effect has been observed in response to negative emotional events where increased autonomic signals enhanced detection and subjective emotional intensity of negative emotional information [81]. Interestingly, false feedback on increased cardiac rate has also been found to heighten the subjective emotional intensity of neutral stimuli [92]. These findings suggest that afferent autonomic information that carries physiological arousal information to aINS mediates the perceived salience and emotional intensity of external stimuli [77, 90]. Therefore, PAG-inhibition of aINS likely attenuates physiological arousal information in the aINS during negative emotional stimuli processing [93]. A lack of PAG-inhibition of aINS in the clinical group likely results in an increased impact of interoceptive/autonomic signalling during negative emotional processing, contributing to heightened salience attribution and affective responses to negative emotional stimuli [90, 94, 95]. This interpretation is consistent with the failure of interoceptive inference forwarded in computational psychiatry as a mechanistic explanation for mood and anxiety disorders [35, 96].

Significant group differences also emerged in average self-inhibitory connectivity of the PAG, which was increased in the clinical group. The strength of this connectivity predicted the diagnostic status of participants. Increased self-inhibition of the PAG could reflect a disrupted inhibitory-excitatory balance within the PAG, thereby decreasing gain control – the sensitivity of the PAG to the inputs from the rest of the network [62]. Indeed, within-PAG activity is governed by mutual inhibitory/excitatory influences between ventral and dorsal subregions that enables flexible sensorimotor and autonomic responses to contextual changes [19, 97], which may be impaired in mood and anxiety disorders [98]. Supporting this, previous animal work has identified decreased excitatory (glutamatergic) activity within the ventral PAG in chronic stress-induced depression [50, 51]. In a similar line, altered intrinsic connectivity of PAG subregions has been identified in post-traumatic stress disorder [99]. Increased self-inhibition of the PAG in clinical participants is indicative of increased stress exposure and high allostatic-load common to aetiology of mood and anxiety disorders [100, 101]. Reduced PAG sensitivity to inputs from the SN likely also underscores the decreased excitatory modulation we observed in amygdala-to-PAG connectivity. As noted above, amygdala input to PAG is integral in driving adaptive PAG-mediated responses upon contextual changes, such as detecting threat [14, 19]. A lack of PAG responsivity to SN inputs would lead to disruptions in engaging adaptive visceromotor and autonomic responses to negative emotional events [14, 94].

### The bottom-up amygdala modulation of the SN did not differ between clinical and healthy control participants

Contrary to our hypothesis, we did not find group differences in bottom-up connectivity from the amygdala. Although increased amygdala reactivity to negative emotional information has been commonly observed in mood and anxiety disorders [4, 5], other work has also reported no change in amygdala activity during negative emotion processing [102] or disorder-specific alterations such as for generalized anxiety disorder [103]. For instance, reduced amygdala connectivity with SN has been previously observed in generalized anxiety disorder [103]. Our clinical sample featured a large portion of participants with generalized anxiety disorder (*n* = 18), suggesting that clinical heterogeneity in our sample may have impacted this amygdala finding.

### Limitations & conclusion

This study featured a transdiagnostic sample with high levels of co-morbidity and most participants were young and female. Epidemiological evidence suggests that mood and anxiety disorders present with high levels of co-morbidity and are more prevalent in females [104], supporting the external validity of our findings. However, sex differences in connectivity have been reported for our regions of interest [67, 94] and replicating our findings in a more balanced sample is warranted. Moreover, although using a transdiagnostic sample allowed us to examine impairments in subcortical-cortical interactions common to frequently comorbid mood and anxiety disorders, disorder-specific effects may have been present. These could be teased apart in future work using discrete diagnostic samples.

Expanding upon previous animal and human neuroimaging work [27, 49, 51], the current findings provide novel evidence for aberrant PAG effective connectivity during negative emotion processing in mood and anxiety disorders. Specifically, the lack of inhibitory PAG modulation of aINS activity in clinical participants may have a role in biased salience attribution and enhanced affective responses to negative emotional events commonly observed in mood and anxiety disorders [1, 5]. The size of this group effect was large enough to predict diagnostic status, negative affect, depression and stress symptoms. Overall, our findings highlight that the processing of negative emotional stimuli is supported by bidirectional interactions across a distributed set of brain regions and provide evidence that the neural basis of impairments in this circuitry can exist at the level of subcortical brain regions such as the PAG in mood and anxiety disorders.

## Supplementary information

Supplementary Information

## Data Availability

Data and code for effective connectivity analysis and group-level GLM results presented here are available at https://github.com/Sevilince/Clinical_Subcortical_SN_DCM.git.
