# Supplementary material for: Subcortical modulation of the salience network during negative emotional processing in mood and anxiety disorders
Source: Mol Psychiatry. 2025 Aug 28;30(11):5475–85. doi: 10.1038/s41380-025-03135-5 (PMC12532698; doi:10.1038/s41380-025-03135-5)
Supplement: Supplementary file 1 — Supplementary Information [file 41380_2025_3135_MOESM1_ESM.docx]

**Subcortical modulation of the salience network during negative emotional processing in mood and anxiety disorders**

Sevil Ince^1,2^, Ben J. Harrison^2^, Kim L. Felmingham^1^, Alec J. Jamieson^2^, Christopher G. Davey^2^, James A. Agathos^2^, Bradford A. Moffat^3^, Rebecca K. Glarin^3^, Trevor Steward^1,2^

^1^ Melbourne School of Psychological Sciences, The University of Melbourne, Parkville, Victoria, 3010, Australia

^2^ Department of Psychiatry, The University of Melbourne, Parkville, Victoria, 3010, Australia

^3^ The Melbourne Brain Centre Imaging Unit, Department of Radiology, The

University of Melbourne, Parkville, Victoria, 3010, Australia

**Content:** The supplementary information file contains supplementary methods (detailing experimental paradigm, image acquisition and pre-processing, general linear modelling and timeseries extraction for dynamic causal modelling), all supplementary tables (S1-S11), and figures (S1-S4).

# **Supplementary Methods**

## **Experimental Paradigm**

Participants completed the emotional oddball paradigm as detailed in Ince and colleagues (1). Briefly, participants were asked to identify a ‘target’ image in a stream frequently presented ‘standard’ image, and infrequent negative emotional and neutral distractor images. The standard image was of neutral valence and presented for the majority of trials (240 presentations, 80% of all trials) while the remaining trials consisted of infrequent oddball presentations (20 trials for each oddball category- target, negative emotional and neutral oddballs). Among these oddball images, the target was a single image of neutral valence. At the beginning of the task, participants were shown this target image and instructed to count the presentations of it throughout the task. Remaining oddball trials consisted of trial-unique novel images with neutral valence (neutral oddballs) and novel images with negative emotional valence (negative emotional oddballs). All images were shown for 800 ms with an interstimulus interval of 1900 ms ± 300 ms. All oddball trials were randomly intermixed the standard image trials and the order of consecutive oddball presentations was random to prevent the predictability of the target image presentations. Furthermore, the successive oddballs were separated by an interval of approximately 10-15s to allow for hemodynamic response to return to baseline. Images were sourced from the Nencki Affective Picture System (NAPS; (2).The NAPS image categories used in the paradigm and their respective IDs are reported in Table S2. Lower-perceptual features of the images (e.g., luminance, complexity, contrast) were not significantly different across image categories. The oddball task was programmed using E-Prime 3.0 (3) and presented on a 32’ LCD BOLD screen (Cambridge Research Systems), which was projected to a reverse mirror mounted to the participants’ head coil during scanning. To determine whether participants sufficiently paid attention during the task, a threshold of at least 75% of true target oddball count (i.e., 15 out of 20) was used, and participants were excluded if they reported an oddball count below this threshold. Upon completion of the scanning session, participant ratings on arousal and valence of each image were collected on a 9-point Likert scale. To compare participants’ post-scan arousal and valence responses, independent samples t-tests and repeated measures ANOVA were conducted in IBM SPSS Statistics (version 29).

**Image acquisition**

Image acquisition was performed on a 7-Tesla research scanner (Siemens Healthcare, Erlangen, Germany) equipped with a 32-channel head coil (Nova Medical Inc., Wilmington MA, USA). Functional (T2^*^-weighted) images (CMRR; (4) were obtained using a multi-band and grappa accelerated gradient-echo planar imaging (GE-EPI) sequence in the steady state (multi-band factor =6, parallel imaging factor =2, repetition time (TR) =800 ms, echo time =22.2 ms, and flip angle =45°, in a 208 cm field-of-view, with 130 ×130 pixel matrix, in-plane voxel size =1.6 ×1.6 mm) with 84 interleaved slices of 1.6mm thickness (no gap) parallel to the anterior-posterior commissure line, covering the whole brain. The total sequence time was approximately 13 mins for the emotional oddball task, resulting in 1020 whole-brain EPI volumes. A high resolution structural T1-weighted image was obtained from each participant using magnetization-prepared 2 rapid gradient echo sequence (MP2RAGE) for co-registration with the functional images (parallel imaging factor = 4, repetition time = 5 seconds, echo time = 2.04 ms, flip angle = 13°; in a 24 cm field of view, with a 320 ×320–pixel matrix, in-plane voxel size = 0.75 ×0.75 mm, slice thickness = 0.75 mm with 224 sagittal slices aligned parallel to the midline). To minimise head movement during scanning, foam pads were inserted to the either side of participants’ heads. During the session, respiration and cardiac pulse were also recorded at 50 Hertz (Hz) and 200 Hz, respectively, via a Bluetooth respiratory belt and pulse-oximeter (Siemens) to be used for physiological noise correction (see section *Pre-processing*).

**Image analysis**

**Pre-processing**

Data pre-processing and analysis were conducted using Statistical Parametric Mapping (SPM) 12 (v7771; (5) software implemented within a MATLAB environment (6); Version 9.6, 2023a). Participants’ time-series were realigned to the mean functional image using least-squares minimization and a six-parameter rigid-body spatial transformation. Resultant realignment parameters were imported to Motion-fingerprint toolbox (7) to assess scan-to-scan head motion for each participant. Participants were excluded if mean total scan-to-scan displacement exceeded 2 mm. Each participant’s structural image was co-registered to their mean functional image from realignment, then segmented and normalized into an International Consortium for Brain Mapping (ICBM152) template using unified segmentation and the DARTEL toolbox (8). Normalized functional images were smoothed with a 3.2-mm full-width-at-half-maximum (FWHM) Gaussian kernel to preserve spatial specificity.

To model physiological noise, cardiac and respiratory recordings were imported to PhysIO toolbox (9), in which physiological noise models were applied to these recordings (i.e., the retrospective image-based correction for a third order cardiac, fourth order respiratory, and first order interaction Fourier expansion of cardiac and respiratory phase (10), respiratory volume per time, (11)

heart rate variability response (12)). Further nuisance regressors were obtained for BOLD signals in white matter (WM) and cerebrospinal fluid (CSF). For this, normalized WM and CSF tissue segmentation from each participant’s structural image were used as noise regions of interest. From these noise regions of interest, mean WM and CSF timeseries and principal components were derived using CompCor (13) as implemented in PhysIO toolbox (9).These regressors were added to six movement regressors derived from realignment and taken to the first-level general linear model to remove variances of no-interest.

**General linear modelling (GLM)**

Pre-processed timeseries were entered into a first-level SPM GLM where trial onsets (standards, targets, negative emotional and neutral oddballs) were convolved with canonical hemodynamic response function and included in the model as the regressors of interest. Interstimulus interval periods served as the implicit baseline. The abovementioned nuisance regressors were included in the model to account for motion and physiological noise. Timeseries were high-pass filtered with a cut-off period of 128s to account for low-frequency noise. Temporal autocorrelations were modelled using FAST, which has been found to perform better than AR(1) for short TRs at 7T (14). As per our previous study (1), the primary contrast of interest at the first level was the direct comparison of negative emotional and neutral oddballs (NEG *>* NEU) to identify changes in brain activation associated with negative emotional saliency response. Contrast images were estimated for each participant and entered to a second-level random-effects GLM using one sample t-test, which was corrected for multiple comparisons using a whole-brain, voxel-wise false discovery rate (FDR) threshold (*p<*.05, K_E_ ≥10).

**Dynamic causal modelling (DCM)**

**Model space and timeseries extraction**

The candidate model space consisted of five regions of interest (ROI); the PAG, amygdala, aINS, dACC and vlPFC. For timeseries extraction and to specify conditions for our model space, a separate GLM (other than the one specified for group-level GLM analysis) was estimated at the first level for three conditions; task (single regressor including the onsets of standards, negative emotional and neutral oddballs), negative emotional salience (single regressor onsets of negative emotional oddballs) and targets (single regressor onsets of targets). Targets were not included in the further DCM analysis. Representative timeseries were extracted from each ROI at the subject level following recently published guidelines (15). Firstly, the centre coordinates of the ROIs were identified by the subject-specific local maxima for the negative emotional salience condition. For the amygdala, aINS, dACC, and vlPFC these coordinates were constrained to be within the 8mm of the group-level peak GLM result for NEG>NEU contrast (see Table S6). A principle eigenvariate from each ROI was extracted using all active voxels (at a threshold of *p.<*05, uncorrected) within a sphere with a 4mm radius from the ROIs’ centre. For the amygdala and vlPFC ROI, inclusive amygdala and vLPFC masks (16) were applied during timeseries extraction. This was done to prevent capturing of voxels from adjacent regions, such as insula which lies in close proximity to the vlPFC. Due to the anatomical size of the PAG and its proximity to nearby midbrain regions, the PAG timeseries were extracted from all active voxels (at a threshold of *p.<*05, uncorrected) within a PAG mask (17) to ensure the inclusion of only PAG voxels. This mask was created via diffusion-based segmentation of human PAG and previously validated (18,19). All timeseries were adjusted using an F-contrast to mean-correct. In the case of a subject having activation below the threshold in a ROI or when a subject-specific peak lay beyond 8mm from the group-peak coordinates, the statistical threshold was further lowered to up to *p*<.25, uncorrected, until a peak ROI coordinate was identified as per recommended by recent guidelines (15). In doing so, we excluded one participant who had their subject-specific local maxima in the aINS ROI (8mm) beyond the group-level peak GLM result for NEG>NEU contrast.

# **Supplementary Tables**

**Table S1. Summary of diagnosis and comorbid DSM-V Axis I disorders in clinical**

**participants**

|  | ***n*** | ***%*** |
| --- | --- | --- |
| Comorbid mood and anxiety disorders | 22 | 59.5 |
| Comorbidities -Any | 30 | 81.1 |
| Mood disorders |  |  |
| Major depressive disorder | 16 | 43.2 |
| Persistent depressive disorder | 13 | 35.1 |
| Pre-menstrual depressive disorder | 1 | 2.7 |
| Anxiety disorders |  |  |
| Generalized anxiety disorder | 18 | 48.6 |
| Social anxiety disorder | 16 | 43.2 |
| Panic disorder | 3 | 8.1 |
| Specific phobia | 3 | 8.1 |
| Trauma and stress related disorders |  |  |
| Post-traumatic stress disorder | 7 | 18.9 |
| Adjustment disorder with anxiety | 1 | 2.7 |
| Eating disorders |  |  |
| Anorexia nervosa | 1 | 2.7 |
| Bulimia nervosa | 1 | 2.7 |
| Binge eating disorder | 1 | 2.7 |

**Table S2. NAPS images used in the emotional oddball paradigm**

| **Stimulus category** | **Negative emotional oddball** | **Neutral oddball** | **Target** | **Standard** |
| --- | --- | --- | --- | --- |
| **NAPS Image category and ID** | Faces_366 | Objects_130 | Objects_299 | Landscapes_057 |
|  | People_023 | People_150 |  |  |
|  | Animals_073 | People_023 |  |  |
|  | People_220 | Animals_073 |  |  |
|  | People_202 | Objects_237 |  |  |
|  | People-017 | People_220 |  |  |
|  | Faces_293 | Faces_194 |  |  |
|  | People_208 | People_202 |  |  |
|  | People_240 | Objects_204 |  |  |
|  | Animals_012 | Landscapes_076 |  |  |
|  | Faces_283 | Faces_305 |  |  |
|  | Objects_139 | People-017 |  |  |
|  | People_198 | Faces_293 |  |  |
|  | People_127 | People_208 |  |  |
|  | People_001 | Objects_230 |  |  |
|  | Faces_174 | Landscapes_091 |  |  |
|  | People_020 | People_240 |  |  |
|  | Faces_367 | Objects_247 |  |  |
|  | Landscapes_007 | Animals_012 |  |  |
|  | Animals_042 | Objects_196 |  |  |

Note. NAPS; Nencki Affective Picture System (2).

**Table S3. Skewness of total and subscale DASS scores**

|  | *W* | *p* Value |
| --- | --- | --- |
| DASS Total | *.922* | <.001 |
| DASS Depression | .872 | <.001 |
| DASS Anxiety | .877 | <.001 |
| DASS Stress | .960 | <.001 |

Note. Skewness tested using Shapiro-Wilk (*W*)*.* DASS, Depression Anxiety and Stress Scale (20,21).

**Table S4. Task performance and post-scan arousal and valence ratings for negative and neutral oddball across healthy control and clinical participants**

|  | mean (*SD*) | | | | *t* statistic ^1^ | | *p* value | Effect size (*d*) |
| --- | --- | --- | --- | --- | --- | --- | --- | --- |
|  | HC, n=37 | | Clinical, n=37 | |  | |  |  |
| Movement During Task | 0.99(.44) | | 0.98(.45) | | *t*_72_=.08 | | .936 | .19 |
| Oddball Count | 19.62(.95) | | 19.84(1.24) | | *t*_72_=-.84 | | .402 | -.20 |
| Negative Oddball Valence | 2.78(.55) | | 2.74(.57) | | *t*_72_=.31 | | .759 | .07 |
| Negative Oddball Arousal | 6.52(.91) | | 6.62(.66) | | *t*_72_=-.51 | | .614 | -.12 |
| Neutral Oddball Valence | 5.09(.39) | | 5.08(.25) | | *t*_72_=.25 | | .804 | .06 |
| Neutral Oddball Arousal | 4.27(1.01) | 4.43(0.93) | | | | *t*_72_=-.69 | .494 | -.16 |
|  |  |  | | *F statistic* ^2^ | | | *p* value | Effect size (partial *η^2^*) |
| Negative Emotional vs Neutral Oddball Valence | | | | *F_(1,72)_=*917.06 | | | <.001 | .93 |
| Diagnostic Group (HC vs clinical) | | | | *F_(1,72)_=*.16 | | | .690 | .02 |
| Diagnostic Group*Valence | | | | *F_(1,72)_=*.03 | | | .891 | .00 |
| Negative Emotional vs Neutral Oddball Arousal | | | | *F_(1,72_*_)_=222.72 | | | <.001 | .76 |
| Diagnostic Group (HC vs clinical) | | | | *F_(1,72)_=.76* | | | .388 | .10 |
| Diagnostic Group*Arousal | | | | *F_(1,72)_=.04* | | | .837 | .001 |

Note. HC, healthy control participants. Participant ratings on arousal and valence of negative and neutral oddballs were collected on a 9-point Likert scale upon completion of the scanning session.

^1^ Independent sample t-test statistics assessing group differences on post-scan arousal and valence ratings for negative emotional and neutral oddballs.

^2^ F statistics results from the two-way repeated measures ANOVA with post-scan arousal and valence ratings for negative emotional and neutral oddballs as within-subjects factor and diagnostic group as between-subjects factor.

**Table S5. Significant activations associated with the negative emotional salience**

| Cluster size (1.6 mm^3^ voxels) | Brain region | Peak  *t-*value | Peak MNI coordinates | | | Side |
| --- | --- | --- | --- | --- | --- | --- |
|  |  |  | x | y | z |  |
| 7030 | Fusiform Gyrus | 10.33 | 38 | -48 | -18 | R |
|  | Middle temporal gyrus | 8.70 | 48 | -56 | 8 | R |
|  | Middle temporal gyrus | 8.04 | 48 | -64 | 10 | R |
| 19435 | Amygdala | 9.73 | 22 | -3 | -18 | R |
|  | Periaqueductal Gray | 9.27 | 2 | -32 | -8 | R |
|  | Insula | 8.94 | -29 | 14 | -14 | L |
| 7416 | Fusiform Gyrus | 9.53 | -37 | -48 | -18 | L |
|  | Middle temporal gyrus | 8.08 | -48 | -61 | 8 | L |
|  | Middle Occipital gyrus | 7.88 | -37 | -67 | 18 | L |
| 8855 | Anterior cingulate cortex, supracallosal | 7.95 | 0 | 13 | 24 | - |
|  | Middle cingulate &  paracingulate gyri | 6.97 | 3 | 16 | 35 | R |
|  | Supplementary motor area | 6.84 | 8 | 11 | 66 | R |
| 503 | Parietal Superior | 5.98 | -29 | -51 | 59 | L |
|  | Parietal Inferior^1^ | 4.55 | -22 | -46 | 46 | L |
|  | Parietal Superior | 3.86 | -37 | -50 | 67 | L |
| 1125 | SupraMarginal gyrus | 5.97 | -64 | -26 | 32 | L |
|  | SupraMarginal gyrus | 5.15 | -54 | -26 | 34 | L |
|  | SupraMarginal gyrus | 4.35 | -62 | -34 | 30 | L |
| 1323 | SupraMarginal gyrus | 5.90 | 59 | -24 | 35 | R |
|  | SupraMarginal gyrus | 5.58 | 59 | -22 | 26 | R |
|  | Postcentral gyrus | 4.39 | 64 | -16 | 40 | R |
| 246 | Superior temporal gyrus | 5.24 | 46 | -19 | -11 | R |
|  | Middle temporal gyrus^1^ | 4.37 | 43 | -27 | -8 | R |
|  | Superior temporal gyrus | 2.84 | 56 | -8 | -13 | R |
| 175 | Precuneus | 5.21 | 11 | -46 | 58 | R |
|  | Middle cingulate &  paracingulate gyri | 4.64 | 11 | -43 | 50 | R |
| 807 | Precentral gyrus | 4.96 | -42 | -6 | 46 | L |
|  | Precentral gyrus | 4.79 | -46 | 0 | 29 | L |
|  | Postcentral gyrus | 4.70 | -38 | -14 | 46 | L |
| 237 | Temporal pole: Middle temporal gyrus | 4.93 | 29 | 11 | -42 | R |
|  | Fusiform gyrus | 4.8 | 35 | 0 | -34 | R |
|  | Temporal pole: Superior temporal gyrus | 3.04 | 34 | 11 | -32 | R |
| 525 | Parietal Superior | 4.77 | 29 | -54 | 56 | R |
|  | Parietal Superior | 4.53 | 32 | -59 | 66 | R |
|  | Parietal Superior | 4.33 | 34 | -50 | 67 | R |
| 84 | Inferior temporal gyrus | 4.75 | -40 | -26 | -21 | L |
| 129 | Inferior temporal gyrus | 4.68 | -32 | 5 | -40 | L |
|  | Temporal pole: Middle temporal gyrus | 4.03 | -22 | 8 | -37 | L |
|  | Inferior temporal gyrus | 3.54 | -35 | -2 | -35 | L |
| 54 | Postcentral gyrus^1^ | 4.54 | 35 | -26 | 34 | R |
|  | Postcentral gyrus | 2.98 | 42 | -21 | 37 | R |
| 112 | Lingual gyrus | 4.31 | -16 | -85 | -14 | L |
| 164 | Lobule IX of cerebellar  hemisphere | 4.3 | 5 | -50 | -38 | R |
|  | Lobule IX of vermis | 3.75 | -3 | -58 | -38 | L |
|  | Lobule IX of vermis | 3.09 | 5 | -59 | -35 | R |
| 28 | Precentral gyrus | 4.13 | -56 | 6 | 42 | L |
| 25 | Parahippocampal gyrus | 4.06 | 37 | -14 | -26 | R |
| 18 | Inferior temporal gyrus | 4.05 | 54 | -61 | -21 | R |
| 32 | Temporal pole: Middle temporal gyrus | 3.97 | 54 | 5 | -21 | R |
| 28 | Medial orbital gyrus^1^ | 3.97 | -21 | 29 | -26 | L |
| 82 | Calcarine fissure and  surrounding cortex | 3.96 | -14 | -56 | 5 | L |
|  | Lingual gyrus | 3.13 | -16 | -59 | -3 | L |
| 13 | Lobule VIII of cerebellar  hemisphere | 3.87 | 11 | -70 | -43 | R |
| 42 | Unlabelled | 3.83 | 14 | -14 | -37 | R |
| 120 | Superior frontal gyrus,  dorsolateral | 3.82 | -21 | 45 | 27 | L |
|  | Superior frontal gyrus,  dorsolateral | 3.64 | -26 | 38 | 30 | L |
|  | Middle frontal gyrus | 3.49 | -21 | 37 | 24 | L |
| 14 | Parahippocampal gyrus | 3.82 | 26 | -11 | -38 | R |
| 10 | Lobule IV, V of cerebellar  hemisphere | 3.81 | -5 | -61 | -14 | L |
| 30 | Postcentral gyrus | 3.76 | -42 | -35 | 48 | L |
| 16 | Lobule VI of cerebellar  hemisphere | 3.76 | -24 | -51 | -32 | L |
| 13 | Corpus Callosum (splenium) | 3.75 | -6 | -35 | 19 | L |
| 11 | Lobule VI of cerebellar  hemisphere | 3.73 | 11 | -69 | -21 | R |
| 10 | Corpus Callosum (splenium) | 3.72 | 11 | -32 | 21 | R |
| 21 | Parietal Superior | 3.69 | -18 | -56 | 75 | L |
| 18 | Lobule VIII of cerebellar  hemisphere | 3.62 | 18 | -61 | -46 | R |
| 69 | Parietal Superior | 3.62 | 16 | -62 | 69 | R |
|  | Parietal Superior | 3.24 | 19 | -70 | 64 | R |
|  | Parietal Superior | 2.76 | 24 | -54 | 69 | R |
| 19 | Lobule I, II of vermis | 3.6 | 2 | -43 | -26 | R |
| 10 | SupraMarginal gyrus | 3.57 | -54 | -50 | 27 | L |
| 17 | Medial orbital gyrus^1^ | 3.53 | -10 | 30 | -29 | L |
| 12 | Lobule VIII of cerebellar  hemisphere | 3.51 | -30 | -54 | -51 | L |
| 10 | Lobule VIIB of cerebellar  hemisphere | 3.49 | -8 | -72 | -40 | L |
| 19 | Corpus Callosum (genu) | 3.48 | -11 | 30 | -3 | L |
| 37 | Lobule VIII of cerebellar  hemisphere | 3.48 | -13 | -67 | -45 | L |
| 16 | Posterior cingulate gyrus | 3.47 | -3 | -42 | 18 | L |
| 34 | Middle cingulate &  paracingulate gyri | 3.47 | 16 | -35 | 42 | R |
| 29 | Fusiform gyrus | 3.42 | 40 | -26 | -22 | R |
| 11 | Gyrus rectus | 3.42 | -8 | 37 | -18 | L |
| 11 | Corpus Callosum (splenium) | 3.39 | 13 | -40 | 19 | R |
| 17 | Superior frontal gyrus,  dorsolateral | 3.38 | 27 | -3 | 74 | R |
| 24 | Lobule IX of cerebellar  hemisphere | 3.37 | 11 | -51 | -50 | R |
|  | Lobule IX of cerebellar  hemisphere | 3.03 | 11 | -53 | -42 | R |
| 11 | Unlabelled | 3.34 | -3 | -43 | 6 | L |
| 10 | Unlabelled | 3.27 | 8 | 45 | 59 | R |
| 15 | Medial orbital gyrus | 3.25 | 14 | 14 | -26 | R |
| 11 | Parietal Superior | 3.19 | -29 | -56 | 70 | L |
| 17 | Postcentral gyrus^1^ | 3.17 | 34 | -19 | 34 | R |
|  | Postcentral gyrus^1^ | 2.82 | 32 | -14 | 40 | R |
| 11 | Parietal Superior | 3.15 | -19 | -75 | 43 | L |
| 22 | Parietal Superior | 3.06 | 18 | -50 | 64 | R |
| 13 | Lingual gyrus | 3.00 | -18 | -66 | -10 | L |
| 10 | Inferior frontal gyrus,  opercular part^1^ | 2.96 | -29 | 6 | 26 | L |

Note. Anatomical regions determined with the Automated Anatomical Labelling Atlas 3 (AAL3; Rolls et al., 2020). Table lists three local maxima at least 8mm apart within each cluster. Comparisons were thresholded at voxel-wise *P*_FDR_ *<* .05, K_E_ ≥ 10. *t* values represent peak activation for each cluster. L=left, R=right.

^1^ Cluster peak coordinates fall outside the defined region, therefore, labelled using the nearest available anatomic region using AAL3.

**Table S6. Location of the GLM group maxima regions of interest included in the DCM model space and their hemispheric counterpart**

| Region of interest | t | Peak MNI coordinates | | | Side |
| --- | --- | --- | --- | --- | --- |
|  |  |  |  |  |  |
|  |  | x | y | z |  |
| Amygdala | 9.73 | 22 | -3 | -18 | R |
| PAG | 9.27 | 2 | -32 | -8 | - |
| aINS | 8.94 | -29 | 14 | -14 | L |
| dACC | 7.95 | -2 | 16 | 21 | - |
| vlPFC | 6.64 | 37 | 29 | -13 | R |
| Region of interest in the opposite hemisphere |  |  |  |  |  |
| Amygdala | 8.48 | -22 | -5 | -16 | L |
| aINS | 7.66 | 30 | 19 | -11 | R |
| vlPFC | 5.85 | -37 | 26 | -13 | L |

Note. GLM group maxima for vlPFC ROI was determined using the binary vlPFC mask from AAL3 Atlas (16). aINS, anterior insula; dACC, dorsal anterior cingulate cortex; PAG, periaqueductal gray; vlPFC, ventrolateral prefrontal cortex; L, left; R, right.

**Table S7. Estimated DCM Parameters for Endogenous, Modulatory, and Driving Connections Common Across Clinical and Healthy Control Participants**

| **Connection** | **Ep (Hz)** | **Cp** | **PP** |
| --- | --- | --- | --- |
| **Endogenous connections^1^**  **(A-matrix)** |  |  |  |
| PAG →PAG | -0.11 | 0.0011 | 1.00* |
| PAG →AMG | 0.87 | 0.0004 | 1.00* |
| PAG → aINS | 0.17 | 0.0003 | 1.00* |
| PAG →dACC | 0.32 | 0.0002 | 1.00* |
| PAG →vlPFC | -0.09 | 0.0002 | 1.00* |
| AMG →PAG | -0.31 | 0.0003 | 1.00* |
| AMG →AMG | -0.19 | 0.0008 | 1.00* |
| AMG → aINS | -0.32 | 0.0002 | 1.00* |
| AMG →dACC | -0.18 | 0.0002 | 1.00* |
| AMG →vlPFC | -0.30 | 0.0003 | 1.00* |
| aINS →PAG | -0.17 | 0.0005 | 1.00* |
| aINS →AMG | 0.00 | 0.0000 | 0.00 |
| aINS → aINS | -0.81 | 0.0014 | 1.00* |
| aINS →dACC | -0.14 | 0.0002 | 1.00* |
| aINS →vlPFC | 0.10 | 0.0003 | 1.00* |
| dACC →PAG | -0.17 | 0.0004 | 1.00* |
| dACC →AMG | -0.21 | 0.0004 | 1.00* |
| dACC → aINS | -0.00 | 0.0000 | 0.00 |
| dACC →dACC | -0.78 | 0.0015 | 1.00* |
| dACC →vlPFC | -0.06 | 0.0002 | 1.00* |
| vlPFC →PAG | 0.54 | 0.0007 | 1.00* |
| vlPFC →AMG | -0.14 | 0.0005 | 1.00* |
| vlPFC → aINS | 0.15 | 0.0003 | 1.00* |
| vlPFC →dACC | 0.09 | 0.0003 | 1.00* |
| vlPFC →vlPFC | -0.92 | 0.0020 | 1.00* |
| **Modulatory connections^2^ (B-matrix)** |  |  |  |
| PAG →AMG | -0.82 | 0.0081 | 1.00* |
| PAG → aINS | -0.00 | 0.0000 | 0.00 |
| PAG →dACC | -0.25 | 0.0063 | 1.00* |
| PAG →vlPFC | 0.39 | 0.0122 | 1.00* |
| AMG →PAG | 4.77 | 0.0400 | 1.00* |
| AMG → aINS | 4.29 | 0.0164 | 1.00* |
| AMG →dACC | 3.50 | 0.0149 | 1.00* |
| AMG →vlPFC | 4.50 | 0.0349 | 1.00* |
| aINS →PAG | 1.41 | 0.0434 | 1.00* |
| aINS →AMG | 2.38 | 0.0173 | 1.00* |
| aINS →vlPFC | 1.38 | 0.0297 | 1.00* |
| dACC →PAG | 2.41 | 0.0312 | 1.00* |
| dACC →AMG | 1.80 | 0.0147 | 1.00* |
| dACC →vlPFC | 0.89 | 0.0311 | 1.00* |
| vlPFC →PAG | 0.71 | 0.0188 | 1.00* |
| vlPFC →AMG | -0.53 | 0.0127 | 1.00* |
| **Driving connection (C-matrix)** |  |  |  |
| *Task* →PAG | -0.54 | 0.0007 | 1.00* |
| *Task* →AMG | 0.30 | 0.0006 | 1.00* |

Note. Driving Task input consisted of Standard, Neutral oddball, and Negative emotional oddball trials. aINS, anterior insula; AMG, amygdala; dACC, dorsal anterior cingulate; DCM, dynamic causal modelling; PAG, periaqueductal gray; vlPFC, ventrolateral prefrontal cortex, Cp; covariance; Ep, expected value; Hz, hertz; PP, posterior probability.

*Posterior probability (PP) exceeding .99 shows a non-zero group effect.

^1^ Endogenous parameters reflect the average between-region and within-region effective connectivity across experimental conditions (context-independent).

^2^ Modulatory parameters reflect context dependent (i.e., negative emotional salience induced) changes in effective connectivity between regions.

**Table S8. Differences in DCM Parameters for Endogenous, Modulatory, and Driving Connections Between Clinical and Healthy Control Participants^1^**

| **Connection** | **Ep (Hz)** | **Cp** | **PP** |
| --- | --- | --- | --- |
| **Endogenous connections^2^**  **(A-matrix)** |  |  |  |
| PAG →PAG | 0.15 | 0.0011 | 1.00* |
| PAG →AMG | -0.00 | 0.0000 | 0.00 |
| PAG → aINS | 0.04 | 0.0003 | 1.00* |
| PAG →dACC | -0.00 | 0.0000 | 0.00 |
| PAG →vlPFC | -0.00 | 0.0000 | 0.00 |
| AMG →PAG | -0.04 | 0.0003 | 1.00* |
| AMG →AMG | -0.07 | 0.0007 | 1.00* |
| AMG → aINS | -0.04 | 0.0002 | 1.00* |
| AMG →dACC | -0.00 | 0.0000 | 0.00 |
| AMG →vlPFC | 0.00 | 0.0000 | 0.00 |
| aINS →PAG | 0.08 | 0.0005 | 1.00* |
| aINS →AMG | -0.06 | 0.0005 | 1.00* |
| aINS → aINS | 0.00 | 0.0000 | 0.00 |
| aINS →dACC | 0.00 | 0.0000 | 0.00 |
| aINS →vlPFC | 0.04 | 0.0003 | 1.00* |
| dACC →PAG | 0.06 | 0.0004 | 1.00* |
| dACC →AMG | -0.08 | 0.0003 | 1.00* |
| dACC → aINS | -0.01 | 0.0003 | 0.51 |
| dACC →dACC | 0.00 | 0.0000 | 0.00 |
| dACC →vlPFC | -0.05 | 0.0002 | 1.00* |
| vlPFC →PAG | -0.03 | 0.0010 | 0.63 |
| vlPFC →AMG | 0.05 | 0.0005 | 1.00* |
| vlPFC → aINS | 0.00 | 0.0000 | 0.00 |
| vlPFC →dACC | -0.02 | 0.0004 | 0.55 |
| vlPFC →vlPFC | -0.00 | 0.0000 | 0.00 |
| **Modulatory connections^3^ (B-matrix)** |  |  |  |
| PAG →AMG | -0.00 | 0.0000 | 0.00 |
| PAG → aINS | 0.34 | 0.0066 | 1.00* |
| PAG →dACC | -0.00 | 0.0000 | 0.00 |
| PAG →vlPFC | -0.00 | 0.0000 | 0.00 |
| AMG →PAG | -0.55 | 0.0355 | 1.00* |
| AMG → aINS | 0.00 | 0.0000 | 0.00 |
| AMG →dACC | 0.13 | 0.0212 | 0.53 |
| AMG →vlPFC | 0.00 | 0.0000 | 0.00 |
| aINS →PAG | -0.19 | 0.0439 | 0.59 |
| aINS →AMG | 0.00 | 0.0000 | 0.00 |
| aINS →vlPFC | -0.00 | 0.0000 | 0.00 |
| dACC →PAG | -0.23 | 0.0464 | 0.61 |
| dACC →AMG | -0.00 | 0.0000 | 0.00 |
| dACC →vlPFC | 0.00 | 0.0000 | 0.00 |
| vlPFC →PAG | 0.00 | 0.0000 | 0.00 |
| vlPFC →AMG | -0.17 | 0.0145 | 0.75 |
| **Driving connection (C-matrix)** |  |  |  |
| *Task* →PAG | -0.00 | 0.0001 | 0.03 |
| *Task* →AMG | 0.00 | 0.0001 | 0.05 |

Note. Driving Task input consisted of Standard, Neutral oddball, and Negative emotional oddball trials. aINS, anterior insula; AMG, amygdala; dACC, dorsal anterior cingulate; DCM, dynamic causal modelling; PAG, periaqueductal gray; vlPFC, ventrolateral prefrontal cortex, Cp; covariance; Ep, expected value; Hz, hertz; PP, posterior probability.

*Posterior probability (PP) exceeding .99 shows a non-zero group effect.

^1^ Clinical participants were coded as 1 and healthy controls were coded as -1 in our model.

^2^ Endogenous parameters reflect the average between-region and within-region effective connectivity across experimental conditions (context-independent).

^3^ Modulatory parameters reflect context dependent (i.e., negative emotional salience induced) changes in effective connectivity between regions.

**Table S9. Estimated DCM Parameters for Endogenous, Modulatory, and Driving Connections for Sex and Age Effects**

|  | **Sex Effect^1^** | | | **Age Effect** | | |
| --- | --- | --- | --- | --- | --- | --- |
| **Connection** | **Ep (Hz)** | **Cp** | **PP** | **Ep (Hz)** | **Cp** | **PP** |
| **Endogenous connections^2^**  **(A-matrix)** |  |  |  |  |  |  |
| PAG →PAG | 0.08 | 0.0016 | 1.00* | 0.00 | 0.0000 | 0.00 |
| PAG →AMG | -0.10 | 0.0006 | 1.00* | 0.00 | 0.0000 | 0.00 |
| PAG → aINS | -0.00 | 0.0000 | 0.00 | -0.01 | 0.0000 | 1.00* |
| PAG →dACC | -0.00 | 0.0000 | 0.00 | -0.00 | 0.0000 | 0.00 |
| PAG →vlPFC | 0.00 | 0.0000 | 0.00 | -0.01 | 0.0000 | 1.00* |
| AMG →PAG | -0.00 | 0.0000 | 0.00 | -0.01 | 0.0000 | 1.00* |
| AMG →AMG | 0.00 | 0.0000 | 0.00 | -0.00 | 0.0000 | 0.00 |
| AMG → aINS | -0.00 | 0.0000 | 0.00 | -0.00 | 0.0000 | 0.00 |
| AMG →dACC | -0.00 | 0.0000 | 0.00 | 0.00 | 0.0000 | 0.00 |
| AMG →vlPFC | -0.03 | 0.0009 | 0.60 | -0.01 | 0.0000 | 1.00* |
| aINS →PAG | 0.08 | 0.0007 | 1.00* | 0.03 | 0.0000 | 1.00* |
| aINS →AMG | 0.00 | 0.0000 | 0.00 | -0.01 | 0.0000 | 1.00* |
| aINS → aINS | -0.14 | 0.0028 | 1.00* | -0.02 | 0.0001 | 1.00* |
| aINS →dACC | 0.06 | 0.0003 | 1.00* | -0.00 | 0.0000 | 0.00 |
| aINS →vlPFC | 0.03 | 0.0009 | 0.58 | 0.01 | 0.0000 | 1.00* |
| dACC →PAG | 0.00 | 0.0000 | 0.00 | 0.00 | 0.0000 | 0.00 |
| dACC →AMG | 0.00 | 0.0000 | 0.00 | 0.00 | 0.0000 | 0.00 |
| dACC → aINS | -0.06 | 0.0003 | 1.00* | -0.00 | 0.0000 | 0.00 |
| dACC →dACC | -0.00 | 0.0000 | 0.00 | 0.01 | 0.0001 | 0.68 |
| dACC →vlPFC | 0.00 | 0.0000 | 0.00 | -0.00 | 0.0000 | 0.00 |
| vlPFC →PAG | -0.00 | 0.0000 | 0.00 | -0.02 | 0.0000 | 1.00* |
| vlPFC →AMG | 0.00 | 0.0000 | 0.00 | 0.00 | 0.0000 | 0.00 |
| vlPFC → aINS | -0.02 | 0.0008 | 0.54 | -0.01 | 0.0000 | 1.00* |
| vlPFC →dACC | -0.08 | 0.0005 | 1.00* | -0.01 | 0.0000 | 1.00* |
| vlPFC →vlPFC | 0.00 | 0.0000 | 0.00 | 0.01 | 0.0001 | 0.65 |
| **Modulatory connections^3^**  **(B-matrix)** |  |  |  |  |  |  |
| PAG →AMG | 0.00 | 0.0000 | 0.00 | 0.03 | 0.0005 | 0.69 |
| PAG → aINS | 0.00 | 0.0000 | 0.00 | -0.05 | 0.0003 | 1.00* |
| PAG →dACC | 0.00 | 0.0000 | 0.00 | 0.00 | 0.0000 | 0.00 |
| PAG →vlPFC | -0.00 | 0.0000 | 0.00 | 0.00 | 0.0000 | 0.00 |
| AMG →PAG | -0.00 | 0.0000 | 0.00 | -0.00 | 0.0000 | 0.00 |
| AMG → aINS | 0.00 | 0.0000 | 0.00 | -0.00 | 0.0000 | 0.00 |
| AMG →dACC | 0.00 | 0.0000 | 0.00 | -0.01 | 0.0006 | 0.38 |
| AMG →vlPFC | -0.00 | 0.0000 | 0.00 | -0.11 | 0.0011 | 1.00* |
| aINS →PAG | -0.98 | 0.0535 | 1.00* | -0.22 | 0.0027 | 1.00* |
| aINS →AMG | 0.00 | 0.0000 | 0.00 | -0.00 | 0.0000 | 0.00 |
| aINS →vlPFC | 0.00 | 0.0000 | 0.00 | -0.00 | 0.0000 | 0.00 |
| dACC →PAG | 0.00 | 0.0000 | 0.00 | 0.00 | 0.0000 | 0.00 |
| dACC →AMG | -0.36 | 0.0585 | 0.81 | -0.06 | 0.0010 | 1.00* |
| dACC →vlPFC | 0.00 | 0.0000 | 0.00 | -0.12 | 0.0013 | 1.00* |
| vlPFC →PAG | -0.00 | 0.0000 | 0.00 | 0.04 | 0.0016 | 0.65 |
| vlPFC →AMG | 0.00 | 0.0000 | 0.00 | 0.00 | 0.0000 | 00.00 |
| **Driving connection (C-matrix)** |  |  |  |  |  |  |
| *Task* →PAG | -0.00 | 0.0001 | 0.04 | 0.00 | 0.0001 | 0.08 |
| *Task* →AMG | -0.00 | 0.0001 | 0.03 | -0.00 | 0.0001 | 0.03 |

Note. Driving Task input consisted of Standard, Neutral oddball, and Negative emotional oddball trials. aINS, anterior insula; AMG, amygdala; dACC, dorsal anterior cingulate; DCM, dynamic causal modelling; PAG, periaqueductal gray; vlPFC, ventrolateral prefrontal cortex, Cp; covariance; Ep, expected value; Hz, hertz; PP, posterior probability.

*Posterior probability (PP) exceeding .99 shows a non-zero group effect.

^1^ Female was coded as 1 and male was coded as -1 in our model.

^2^ Endogenous parameters reflect the average between-region and within-region effective connectivity across experimental conditions (context-independent).

^3^ Modulatory parameters reflect context dependent (i.e., negative emotional salience induced) changes in effective connectivity between regions.

**Table S10. Out-of-samples Correlation for Predicted and Observed Diagnostic Status, and Total DASS and DASS Subscale Scores Using Leave-one-out Cross-validation and the Parameters of Interest -Modulatory**

| Connectivity |  | *r* | *p* Value |
| --- | --- | --- | --- |
| PAG→AINS | Group | 0.31* | 0.00342 |
|  | SQRT DASS Total | 0.25* | 0.01731 |
|  | SQRT DASS Depression | 0.31* | 0.00341 |
|  | SQRT DASS Anxiety | 0.09 | 0.23277 |
|  | SQRT DASS Stress | 0.20* | 0.04608 |
|  | DASS Total | 0.30* | 0.00476 |
|  | DASS Depression | 0.34* | 0.00141 |
|  | DASS Anxiety | 0.09 | 0.21252 |
|  | DASS Stress | 0.25* | 0.01790 |
| AMG→PAG | Group | 0.08 | 0.26012 |
|  | SQRT DASS Total | -0.40 | 0.99981 |
|  | SQRT DASS Depression | -0.24 | 0.97771 |
|  | SQRT DASS Anxiety | -0.07 | 0.71158 |
|  | SQRT DASS Stress | -0.26 | 0.98723 |
|  | DASS Total | -0.45 | 0.99997 |
|  | DASS Depression | -0.27 | 0.98857 |
|  | DASS Anxiety | 0.08 | 0.26185 |
|  | DASS Stress | -0.18 | 0.93931 |

Note. **p*<.05. Mean-centred SQRT and standardized mean-centred DASS scores. DASS, Depression Anxiety and Stress Scale (20,21). SQRT, square root transformed values. AMG, amygdala; PAG, periaqueductal gray.

**Table S11. Out-of-samples Correlation for Predicted and Observed Diagnostic Status, and Total DASS and DASS Subscale Scores Using Leave-one-out Cross-validation and the Parameters of Interest -Intrinsic**

| Connectivity |  | *r* | *p* Value |
| --- | --- | --- | --- |
| PAG→PAG | Group | 0.23* | 0.02726 |
|  | SQRT DASS Total | 0.05 | 0.34626 |
|  | SQRT DASS Depression | 0.17 | 0.07365 |
|  | SQRT DASS Anxiety | -0.39 | 0.99962 |
|  | SQRT DASS Stress | -0.00 | 0.50527 |
| PAG→AINS | Group | -0.05 | 0.66216 |
|  | SQRT DASS Total | 0.04 | 0.35456 |
|  | SQRT DASS Depression | -0.18 | 0.93000 |
|  | SQRT DASS Anxiety | 0.06 | 0.32150 |
|  | SQRT DASS Stress | 0.07 | 0.27600 |
| AMG→PAG | Group | -0.35 | 0.99873 |
|  | SQRT DASS Total | 0.05 | 0.34316 |
|  | SQRT DASS Depression | -0.17 | 0.92200 |
|  | SQRT DASS Anxiety | 0.05 | 0.32940 |
|  | SQRT DASS Stress | 0.13 | 0.14143 |
| AMG→AMG | Group | -0.23 | 0.97657 |
|  | SQRT DASS Total | -0.03 | 0.59000 |
|  | SQRT DASS Depression | -0.38 | 0.99960 |
|  | SQRT DASS Anxiety | 0.03 | 0.41642 |
|  | SQRT DASS Stress | 0.01 | 0.46500 |
| AMG→aINS | Group | 0.10 | 0.19668 |
|  | SQRT DASS Total | -0.15 | 0.90155 |
|  | SQRT DASS Depression | -0.35 | 0.99865 |
|  | SQRT DASS Anxiety | 0.01 | 0.48170 |
|  | SQRT DASS Stress | -0.15 | 0.89040 |
| aINS→PAG | Group | 0.02 | 0.41748 |
|  | SQRT DASS Total | -0.48 | 0.99900 |
|  | SQRT DASS Depression | -0.08 | 0.75300 |
|  | SQRT DASS Anxiety | -0.09 | 0.76000 |
|  | SQRT DASS Stress | -0.21 | 0.96073 |
| aINS→AMG | Group | -0.13 | 0.85706 |
|  | SQRT DASS Total | -0.53 | 1.0000 |
|  | SQRT DASS Depression | -0.46 | 0.99990 |
|  | SQRT DASS Anxiety | -0.60 | 1.000 |
|  | SQRT DASS Stress | -0.51 | 1.000 |
| aINS→vlPFC | Group | -0.18 | 0.93230 |
|  | SQRT DASS Total | -0.18 | 0.93230 |
|  | SQRT DASS Depression | -0.21 | 0.96560 |
|  | SQRT DASS Anxiety | -0.21 | 0.96530 |
|  | SQRT DASS Stress | 0.23* | 0.02370 |
| dACC→PAG | Group | 0.13 | 0.13342 |
|  | SQRT DASS Total | 0.20* | 0.04649 |
|  | SQRT DASS Depression | 0.19 | 0.05082 |
|  | SQRT DASS Anxiety | 0.19 | 0.05750 |
|  | SQRT DASS Stress | 0.12 | 0.16100 |
| dACC→AMG | Group | 0.12 | 0.15197 |
|  | SQRT DASS Total | -0.20 | 0.95400 |
|  | SQRT DASS Depression | -0.28 | 0.90000 |
|  | SQRT DASS Anxiety | -0.09 | 0.76970 |
|  | SQRT DASS Stress | -0.15 | 0.90000 |
| dACC→vlPFC | Group | 0.07 | 0.29209 |
|  | SQRT DASS Total | 0.08 | 0.23892 |
|  | SQRT DASS Depression | 0.06 | 0.29864 |
|  | SQRT DASS Anxiety | 0.01 | 0.47000 |
|  | SQRT DASS Stress | 0.08 | 0.24019 |
| vlPFC→AMG | Group | 0.02 | 0.41710 |
|  | SQRT DASS Total | -0.01 | 0.53041 |
|  | SQRT DASS Depression | -0.09 | 0.77000 |
|  | SQRT DASS Anxiety | 0.11 | 0.16896 |
|  | SQRT DASS Stress | 0.05 | 0.34200 |

Note. **p*<.05. Mean-centred SQRT DASS scores. DASS, Depression Anxiety and Stress Scale (20,21). SQRT, square root transformed values. aINS, anterior insula; AMG, amygdala; dACC, dorsal anterior cingulate; PAG, periaqueductal gray; vlPFC, ventrolateral prefrontal cortex.

**Supplementary Figures**

**Figure S1. Emotional oddball paradigm**


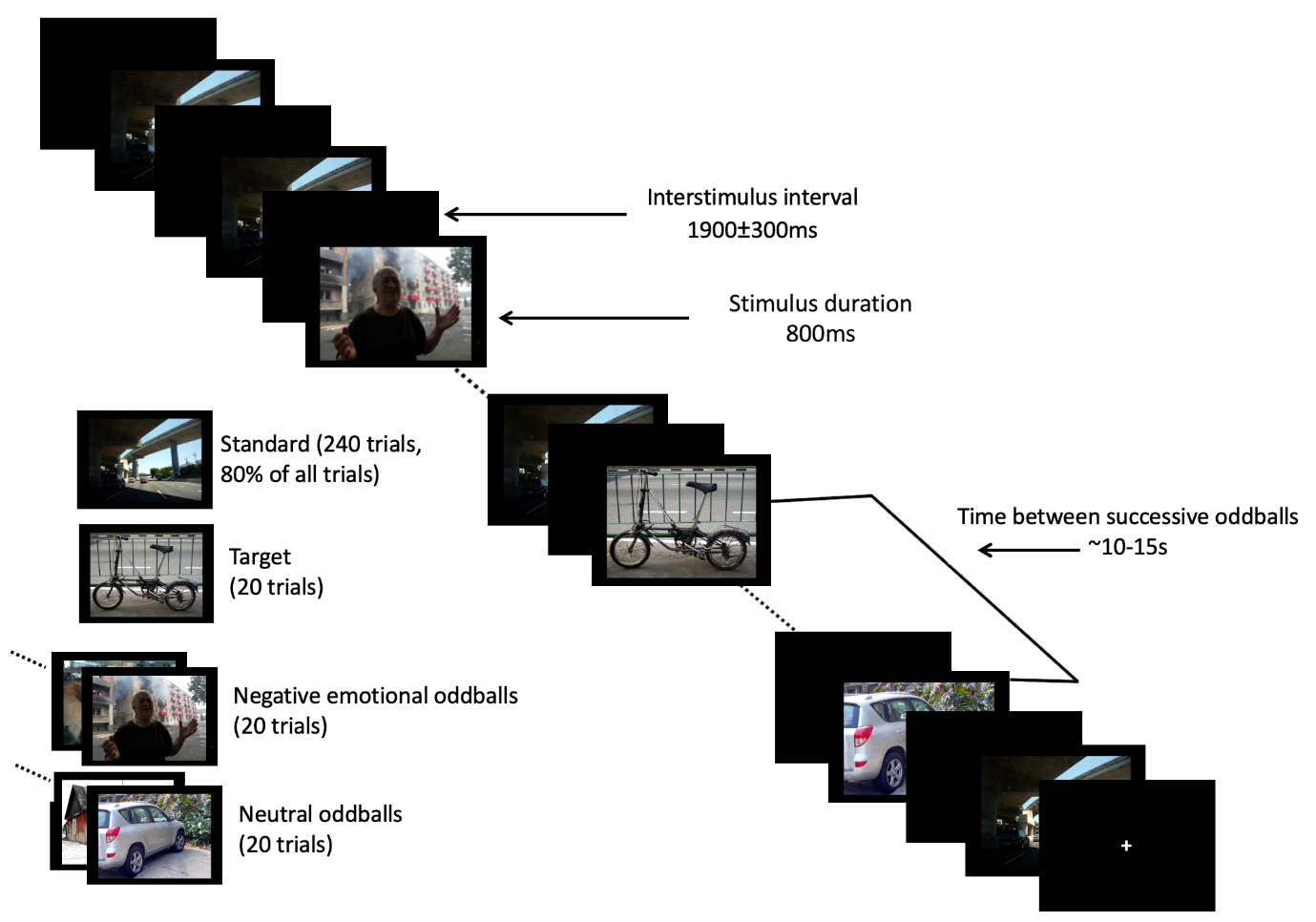


During the task, participants were required to count the number of target oddball image in a string of frequently presented standard images, and neutral and negative oddball images. All oddball images were randomly intermixed with the standard images, with the interval between successive oddballs ranging from approximately 10 to 15 seconds.

**Figure S2. Distribution of the centre coordinates of the VOIs for each subject.**


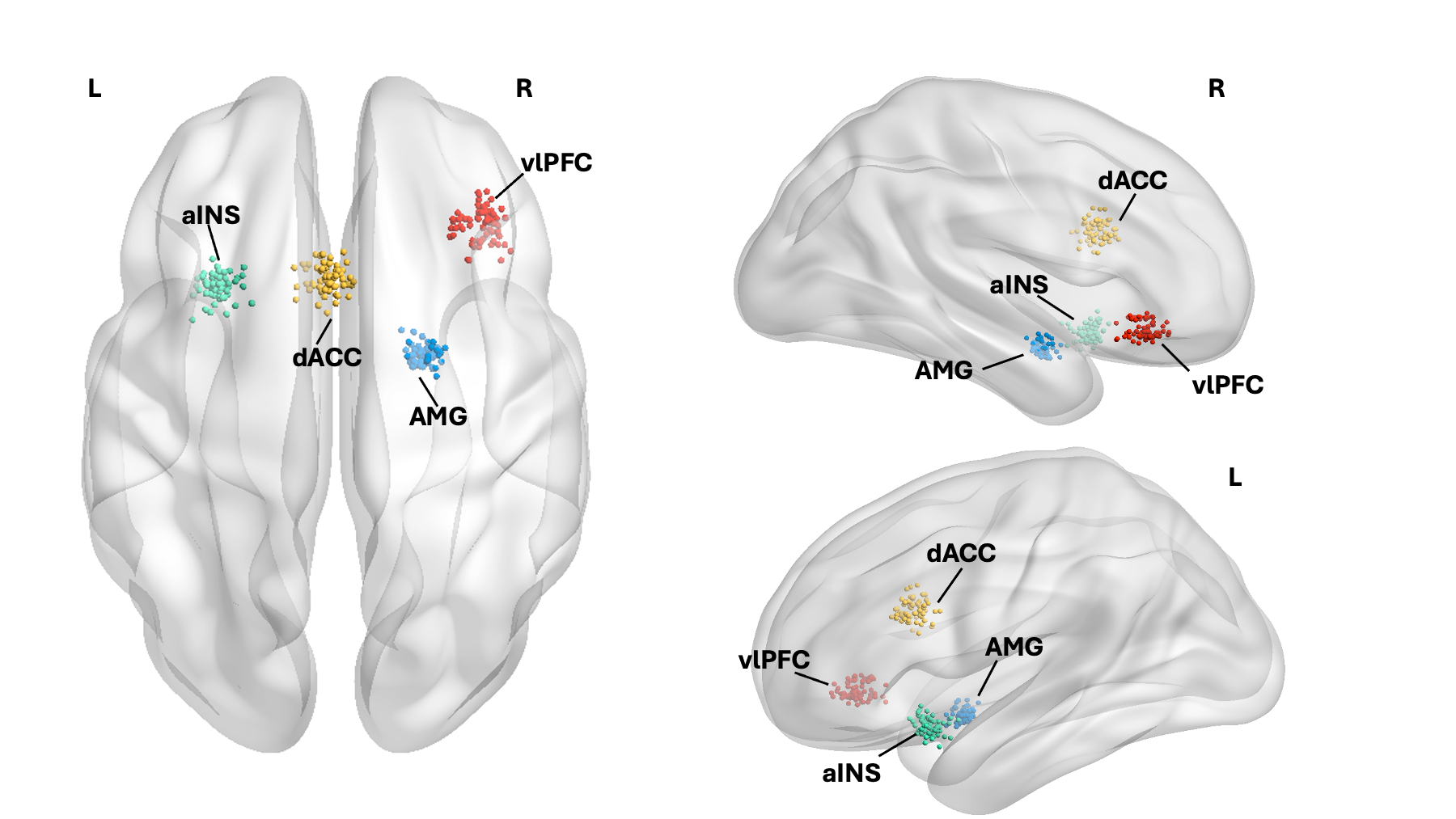


The aINS in turquoise, dACC in yellow, AMG in dark blue and vlPFC in red. Render visualized using Brainnet viewer (22). AMG, amygdala; aINS, anterior insula; dACC, dorsal anterior cingulate; HC, healthy controls; vlPFC, ventrolateral prefrontal cortex, VOI, voxel of interest. L, left; R, right.

**Figure S3. The PAG mask used in the current study**


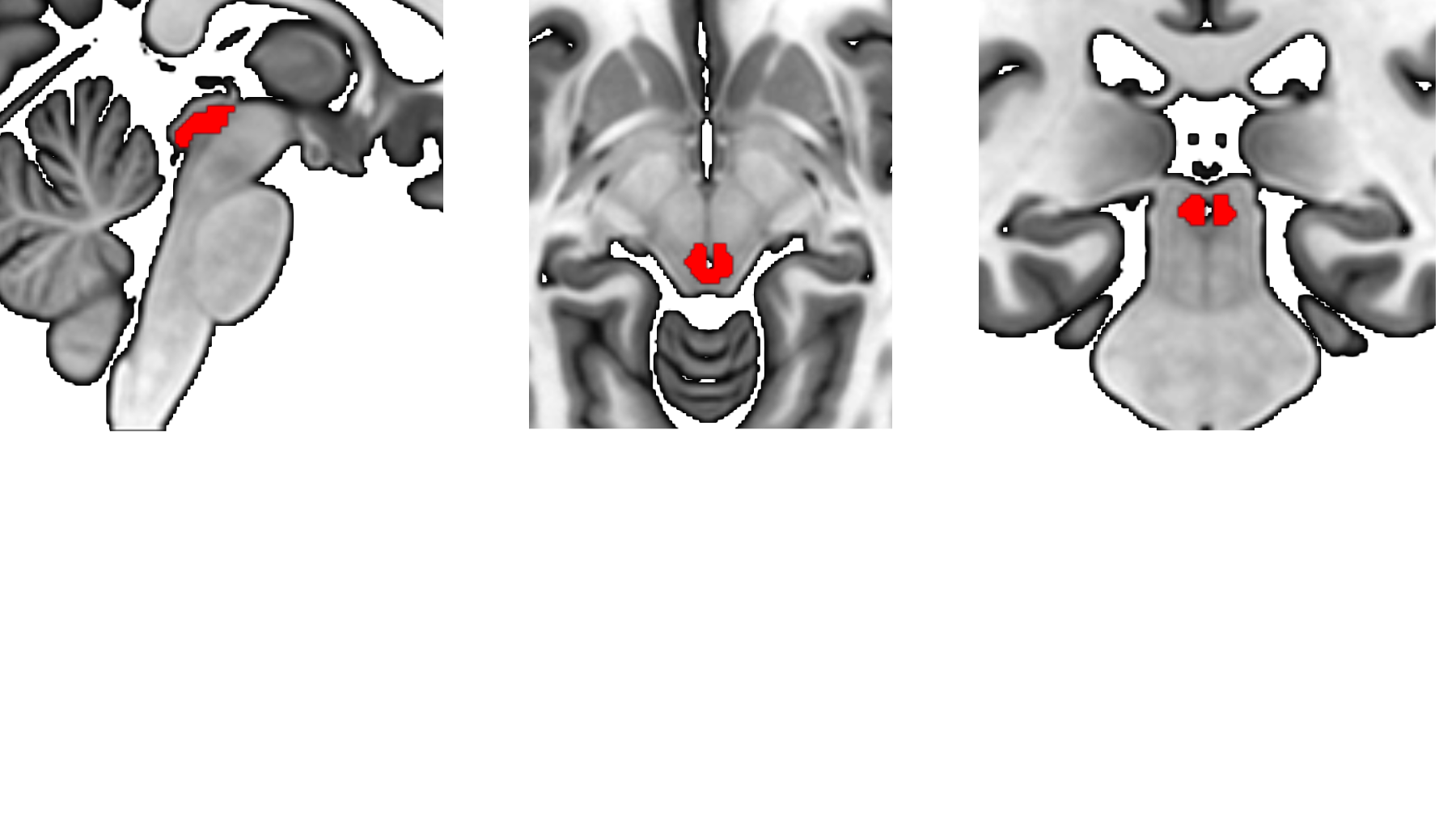


The PAG mask (depicted in red) was obtained from Ezra and colleagues (17) .The mask was created via diffusion-based segmentation of human PAG and previously validated (18,19).

**Figure S4. Leave-one-out cross validation predicting DASS anxiety scores using the modulatory connectivity from PAG to aINS**

**
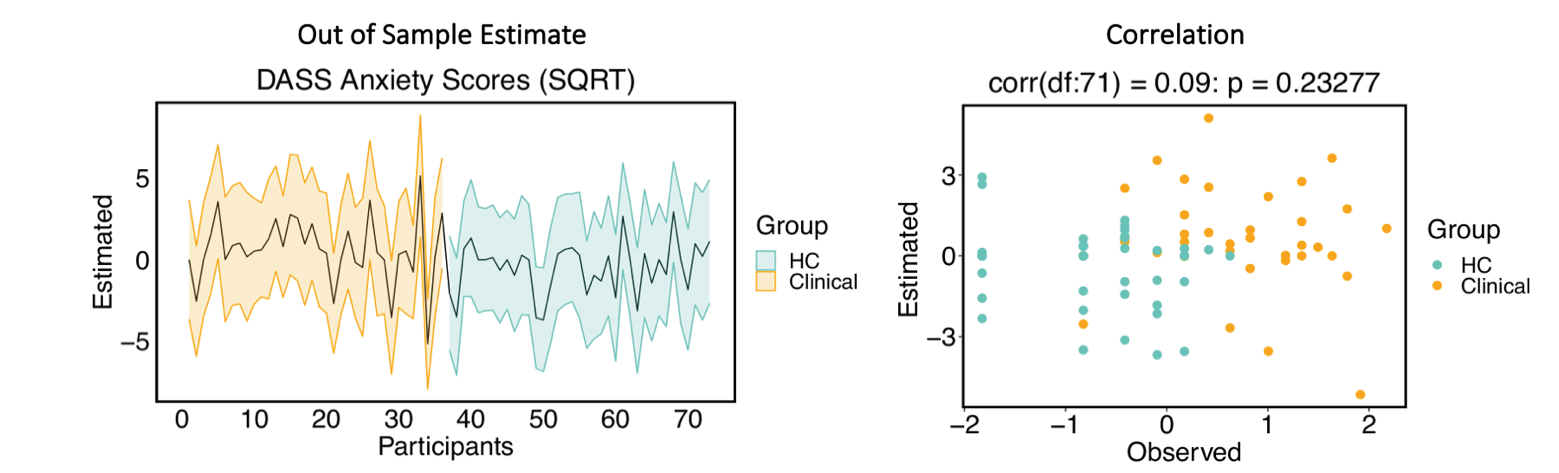
**

Orange depicts clinical and turquoise depicts healthy control (HC) participants. Left: The out of sample estimates (black bold line) across participants with 90% credible interval (shaded area). Right: The correlation between observed and predicted values for mean-centred SQRT DASS anxiety subscale scores. DASS; Depression, Anxiety, Stress Scale. aINS, anterior insula; PAG, periaqueductal gray; SQRT, square root transformed.

**Supplementary References**

1. Ince S, Steward T, Harrison BJ, Jamieson AJ, Davey CG, Agathos JA, et al. Subcortical contributions to salience network functioning during negative emotional processing. Neuroimage. 2023 Apr 15;270.

2. Marchewka A, Żurawski Ł, Jednoróg K, Grabowska A. The Nencki Affective Picture System (NAPS): Introduction to a novel, standardized, wide-range, high-quality, realistic picture database. Behav Res Methods. 2014;46(2):596–610.

3. Psychology Software Tools Inc [E P 3. 0]. Inc., Psychology Software Tools. 2016;Retrieved.

4. Moeller S, Yacoub E, Olman CA, Auerbach E, Strupp J, Harel N, et al. Multiband multislice GE-EPI at 7 tesla, with 16-fold acceleration using partial parallel imaging with application to high spatial and temporal whole-brain FMRI. Magn Reson Med. 2010;63(5):1144–53.

5. The Wellcome Centre for Human Neuroimaging. Statistical Parametric Mapping [Internet]. Available from: https://www.fil.ion.ucl.ac.uk/spm/

6. The Mathworks IncMV 9. 14. MATLAB. www.mathworks.com/products/matlab. Natick, Massachusetts; 2023.

7. Wilke M. An alternative approach towards assessing and accounting for individual motion in fMRI timeseries. Neuroimage. 2012 Feb 1;59(3):2062–72.

8. Ashburner J. A fast diffeomorphic image registration algorithm. Neuroimage. 2007;38(1):95–113.

9. Kasper L, Bollmann S, Diaconescu AO, Hutton C, Heinzle J, Iglesias S, et al. The PhysIO Toolbox for Modeling Physiological Noise in fMRI Data. J Neurosci Methods. 2017;276:56–72; dx.doi.org/10.1016/j.jneumeth.2016.10.019

10. Glover GH, Li TQ, Ress D. Image-Based Method for Retrospective Correction of Physiological Motion Effects in fMRI: RETROICOR. Magnetic Resonance in Medicine: An Official Journal of the International Society for Magnetic Resonance in Medicine. 2000;44(1):162–7.

11. Birn RM, Smith MA, Jones TB, Bandettini PA. The respiration response function: The temporal dynamics of fMRI signal fluctuations related to changes in respiration. Neuroimage. 2008 Apr 1;40(2):644–54.

12. Chang C, Cunningham JP, Glover GH. Influence of heart rate on the BOLD signal: The cardiac response function. Neuroimage. 2009 Feb 1;44(3):857–69.

13. Behzadi Y, Restom K, Liau J, Liu TT. A component based noise correction method (CompCor) for BOLD and perfusion based fMRI. Neuroimage . 2007;37(1):90–101.

14. Olszowy W, Aston J, Rua C, Williams GB. Accurate autocorrelation modeling substantially improves fMRI reliability. Nat Commun. 2019 Dec 1;10(1).

15. Zeidman P, Jafarian A, Corbin N, Seghier ML, Razi A, Price CJ, et al. A guide to group effective connectivity analysis, part 1: First level analysis with DCM for fMRI. Neuroimage. 2019;200:174–90.

16. Rolls ET, Huang CC, Lin CP, Feng J, Joliot M. Automated anatomical labelling atlas 3. Neuroimage . 2020;206(August 2019):116189; doi.org/10.1016/j.neuroimage.2019.116189

17. Ezra M, Faull OK, Jbabdi S, Pattinson KTS. Connectivity-based segmentation of the periaqueductal gray matter in human with brainstem optimized diffusion MRI. Hum Brain Mapp. 2015;36(9):3459–71.

18. Faull OK, Jenkinson M, Ezra M, Pattinson KTS. Conditioned respiratory threat in the subdivisions of the human periaqueductal gray. Elife. 2016;5:1–19.

19. Faull OK, Pattinson KTS. The cortical connectivity of the periaqueductal gray and the conditioned response to the threat of breathlessness. Elife. 2017;6:1–18.

20. Lovibond SH, Lovibond PF. Manual for the Depression Anxiety Stress Scales. 2nd ed. Psychology Foundation of Australia. Sydney: Psychology Foundation of Australia; 1995.

21. Henry JD, Crawford JR. The short-form version of the Depression anxiety stress scales (DASS-21): Construct validity and normative data in a large non-clinical sample. British Journal of Clinical Psychology. 2005 Jun;44(2):227–39.

22. Xia M, Wang J, He Y. BrainNet Viewer: A Network Visualization Tool for Human Brain Connectomics. PLoS One. 2013 Jul 4;8(7).
